# The R2R3MYB Gene Family in *Phyllostachys edulis*: Genome-Wide Analysis and Identification of Stress or Development-Related *R2R3MYBs*

**DOI:** 10.3389/fpls.2018.00738

**Published:** 2018-07-10

**Authors:** Dan Hou, Zhanchao Cheng, Lihua Xie, Xiangyu Li, Juan Li, Shaohua Mu, Jian Gao

**Affiliations:** Key Laboratory of Bamboo and Rattan Science and Technology, State Forestry Administration, International Center for Bamboo and Rattan, Beijing, China

**Keywords:** moso bamboo, R2R3MYB genes, abiotic stress, tissue development, *PheMYB4-1*

## Abstract

The MYB transcription factor (TF) is one of the largest gene families in plants and involved to multiple biological processes. However, little is known about the MYB family and its functional role in the genome of moso bamboo. In the present study, a total of 114 *R2R3MYB* genes were first identified from moso bamboo genome and full-length non-chimeric (FLNC) reads. Phylogenetic analysis coupled with gene structure analysis and motif determination resulted in the division of these *PheR2R3MYB*s into 17 subgroups. The position of eight proteins along an external branch in the phylogenetic tree suggested their relatively ancient origin. The genes in this group were all substituted by (Met, M)/(Arg, R) at conservative W residues in both R2 and R3 repeats, and half were found to possess no transcriptional activation activity. The analysis of evolutionary patterns and divergence suggests that the expansion of PheMYBs was mainly attributable to whole genome duplication (WGD) under different selection pressures. Expressional analysis based on microarray and qRT-PCR data performed diverse expression patterns of *R2R3MYBs* in response to both various abiotic stimuli and flower development. Furthermore, the co-expression analysis of R2R3MYBs suggested an intricate interplay of growth- and stress-related responses. Finally, we found a hub gene, *PheMYB4*, was involved in a complex proteins interaction network. Further functional analysis indicated that ectopic overexpression of its homologous gene, *PheMYB4-1*, could increase tolerance to cold treatment and sensitivity to drought and salt treatment of transgenic *Arabidopsis* seedlings. These findings provide comprehensive insights into the MYB family members in moso bamboo and offer candidate *MYB* genes for further studies on their roles in stress resistance.

## Introduction

The MYB gene family, one of the largest transcription factor (TF) families in plants, is defined by a highly conserved MYB DNA binding domain (DBD) at the N-terminus (Lipsick, 1996). The MYB repeat typically consisted of approximate 50–53 amino acids, each forming three a-helices. “The second and third helices of each repeat build a helix–turn–helix structure with three regularly spaced tryptophan (or hydrophobic) residues, forming a hydrophobic core in the 3D HTH structure” (Ogata et al., 1996). When bind to a promoter sequence, a helix-turn-helix structure intercalates in the major groove, and the third helix of each repeat makes direct contact with a specific DNA sequence motif as a “recognition helix” (Dubos et al., 2010).

In the plant kingdom, MYB proteins are classified into four classes depending on the number of MYB repeats (one, two, three, and four), the most belong to the R2R3-MYB subfamily. Numerous studies have shown that R2R3MYB TF is involve in physiological and biochemical processes, especially in responses to the various biotic and abiotic stresses, and participate in hormone synthesis and signal transduction (Dubos et al., 2010; Du et al., 2012a; Katiyar et al., 2012; Baldoni et al., 2015; Wang et al., 2015), such as *AtMYB15*, *AtMYB30*, *AtMYB60* and *AtMYB96* from *Arabidopsis* (Cominelli et al., 2005; Raffaele et al., 2008; Li et al., 2009; Seo et al., 2009; Seo and Park, 2010), the *OsMYB4*, *OsMYB30*, *OsMYB2*, and *OsMYB3R-2* from rice (Agarwal et al., 2006; Dai et al., 2007; Yang et al., 2012; Lv et al., 2017). Among these, *AtMYB15* is involved in cold and freezing tolerance in *Arabidopsis* by regulating of *CBF* genes, the *myb15* mutants show increased tolerance to freezing stress whereas its overexpression reduces freezing tolerance (Agarwal et al., 2006). *OsMYB4* is strongly induced by cold treatment in rice, its overexpression in *Arabidopsis* significant increased chilling and freezing tolerance of transgenic plants by affecting cold-related genes expressions (Vannini et al., 2004). In rice, *OsMYB30* is another cold-responsive *MYB* gene, overexpression of *OsMYB30* in rice resulted in cold sensitivity increasing while the *osmyb30* mutant showed increased cold tolerance. It is identified that the OsMYB30 should be a novel cold tolerance regulator by interacting with JAZ protein and suppressed the β-*amylase* gene expression (Lv et al., 2017). Recently, the *MYB80* and *MYB124* are found to increase cold and freezing hardiness in apple, by affecting cold-responsive gene expression in both CBF-dependent and CBF-independent pathways (Xie et al., 2018). Several *MYB* genes are involved in regulating the environmental stresses response as well as plant development. An *MYB* gene from *Betula platyphylla* (*BplMYB46*) improved salt and osmotic tolerance of transgenic birch plants. It also increased lignin deposition and secondary cell wall thickness by directly activating the expression of genes involved secondary cell wall biosynthesis (Guo et al., 2017). The overexpression of a *Populus trichocarpa* (*PtrSSR1*) salt-stress-regulator in *Arabidopsis* clearly inhibited lateral root emergence (LRE) and improved salt stress tolerance by integrating the regulation of LRE and abscisic acid (ABA) signaling (Fang et al., 2017). Other functions of *MYBs* include control of cellular morphogenesis, regulation of secondary metabolism, secondary cell wall biosynthesis and meristem formation as key players in plant regulatory network (Nakano et al., 2015; Wang W. et al., 2016; Lloyd et al., 2017). For example, *MaAN2* participates in the control of anthocyanin biosynthesis of *Muscari armeniacum*. It interacted with AtTT8 *in vivo* and strongly activated the promoters of *M. armeniacum* dihydroflavonol 4-reductase (MaDFR) and *M. armeniacum* anthocyanidin synthase (MaANS) (Chen K. et al., 2017). In *Eucalyptus*, “EgMYB1, which is known to repress lignin biosynthesis, interacts specifically with a linker histone variant, EgH1.3. They integrate developmental signals to prevent premature or inappropriate lignification of secondary cell walls, providing a mechanism to fine-tune the differentiation of xylem cells in time and space” (Soler et al., 2017).

China has extensive bamboo resources and a large bamboo industry, and the area of moso bamboo (*Phyllostachys edulis*) forest area accounts for about 74% of the total bamboo forests area. Total bamboo root output is about 4,500,000 tons, providing valuable materials for industrial and agricultural development as well as cultural events in China (Wu and Cao, 2016). Additionally, moso bamboo is characterized by a prolonged vegetative phase before flowering and striking remarkably rapid shoot growth rate (as fast as 1 m/day at peak growth, reaching a height of 20 m in 45–60 days) (Peng et al., 2013a,b; Gao et al., 2014; Li et al., 2016; Ge et al., 2017). During growth and development, moso bamboo is exposed to a variety of environmental that may cause death, severely threatening the bamboo industry (Peng et al., 2013b; Liu et al., 2017). In recent years, a number of gene families involved in the abiotic stress response and plant growth were sequentially identified and analyzed in moso bamboo, such as WRKY, TIFY, IQD and AAAP gene families (Huang et al., 2016; Min et al., 2016; Liu et al., 2017; Long et al., 2017). Peng et al. (2013a) noted that MYB proteins are highly expressed in panicles in comparison to the vegetative tissues of *P. edulis* (Peng et al., 2013a). These proteins were also significantly upregulated during flower development. Moreover, they might be involved in drought-responsive and gibberellic acid (GA)-signaling pathways to improve stress resistance and further activate downstream genes to influence flowering transition (Gao et al., 2014). Although many studies have emphasized the importance of MYB proteins and have facilitate a preliminary understanding of this large gene family, with the exception of *PeMYB2* (Xiao et al., 2013), few members of moso bamboo *MYB* genes have been well functional characterized, particularly in comparison with model plants. There is thus an urgent need to characterize the roles of *MYB*s in moso bamboo and to further our understanding of these genes.

In the current study, we first identified three novel *MYB* genes based on the full-length non-chimeric (FLNC) reads, thereby improving the existing annotation of the genome. The details of R2R3MYB family were also described for the first time, including their gene structures, phylogeny, conserved motifs and *cis*-elements in the promoter sequences. Here, we identified eight genes along the same phylogenetic branch that possessed special gene structures and motifs but had lost transcriptional activation activities. Moreover, the expression pattern of *MYBs* were analyzed during flower development, and a total of 38 MYB TFs were selected and subjected to expression pattern analysis under various abiotic stresses (cold, drought, and salinity). The MYB TFs closely associated with multiple stresses responses were confirmed and the function of *PheMYB4-1* (accession number KU721929.1) was further identified by ectopic overexpression analysis. The co-expression-network-based analysis was also applied to dissect the MYB transcriptional regulatory network and their correlated links in flower development and the abiotic responses of moso bamboo. Our results should inform the characterization of *PheMYB* genes and provide new insights and valuable information for the further identification of this versatile gene family in bamboo.

## Materials and Methods

### Identification of R2R3MYBs in Moso Bamboo and Other Species

For *MYB* gene identification in moso bamboo, multiple database searches were performed. We used the MYB mRNA sequences of *Oryza sativa* and *Zea mays*, obtained from NCBI Nucleotide database^1^, as query sequences to blast against the moso bamboo transcriptome database (downloaded from the bamboo genome database)^2^ (Peng et al., 2013a; Zhao et al., 2014) with the *e*-value cut-off of 10^-5^. The resulting sequences were further subjected to annotation to NCBI non-redundant database with *e*-value cut-off of 10^-10^ using Blast2GO (Conesa et al., 2005; Conesa and Götz, 2008) to confirm the identification, and the wrong annotated sequences were removed (Cao et al., 2016; Deokar and Tar’an, 2016; Sun et al., 2016). Finally, the putative proteins were manually analyzed for the intact MYB domain (PF00249) using the Pfam database^3^ and Conserved Domains Database^4^ with *e*-values < 0.001. Only the gene models containing two or more MYB repeats were considered to belong to the PheR2R3MYB subfamily. The selected MYBs were further screened using the FLNC reads (Wang T. et al., 2017)^5^. To obtain further information of the PheR2R3MYB proteins, grand average of hydropathy (GRAVY), average isoelectric point (PI) value and the molecular weight were predicted by the ProtParam tool^6^. Following this, a total of 135 *Arabidopsis*, 90 rice and 81 *Brachypodium R2R3MYB* genes were sequentially identified (Katiyar et al., 2012). The amino acid sequences of *Arabidopsis* and rice were obtained from The Arabidopsis Information Resource (TAIR) and Rice Genome Annotation Project database^7^^,^^8^ , while the protein sequences of *Brachypodium* R2R3MYBs were download from the Phytozome version 11.0 database^9^.

### Sequences Analysis and Phylogenetic Tree

Multiple sequence alignment was performed using the full-length protein sequences of PheR2R3MYBs in the program ClustalX 2.1. The Clustal Omega^10^ (Sievers et al., 2011) and MUSCLE^11^ (Edgar, 2004a,b) databases were also used to verify the protein sequence alignment results. The neighbor-joining (NJ) and maximum likelihood (ML) phylogenetic trees were constructed using MEGA 7 with 1,000 bootstrap replicates, respectively. To support the subgroup designation of the phylogenetic analysis, the intron patterns of moso bamboo the *R2R3MYB* genes were visualized using the Gene Structure Display Server (GSDS)^12^ by aligning the cDNA sequences to their corresponding genomic DNA sequences. The conserved motifs of the PheR2R3MYBs proteins were defined using the MEME program^13^ (Bailey, 2006; Bailey et al., 2009). The following parameter settings were used: distribution of motifs, zero or one per sequence; maximum number of motifs to find, 50; minimum width of a motif, six; and maximum width of a motif, 250 (to identify long R2R3 domains). Only motifs with an *e*-value of <1e-20 were retained for further analysis (Wang et al., 2015). To analyze the sequence features of the MYB domain of moso bamboo R2R3MYB proteins, the sequences of the R2 and R3 MYB repeats were aligned using ClustalX 2.1. The sequence logos for the R2 and R3 MYB repeats were produced based on the multiple alignment files by the web-based application WebLogo^14^ (Crooks et al., 2004).

### Calculation of *Ka/Ks* Values

To further analyze gene duplication events, the paralogous and orthologous gene-pairs were aligned using ClustalX2.1 and analyzed using DnaSP software to calculation the *Ka* and *Ks* substitution rates (Rozas, 2009). The divergence time (T) was calculated according to T = Ks/(2 × 6.5 × 10^-9^) MYA for moso bamboo, rice, maize and *Brachypodium* (Wu et al., 2016).

### *In silico* Analysis of *Cis*-Acting Elements of PheR2R3MYBs

The elements in the promoter fragments (from -1500 bp to the transcription start site) of the *PheR2R3MYB* genes were analyzed using the program PlantCARE online^15^ (Rombauts et al., 1999; Lescot et al., 2002).

### Plant Materials and Treatments

The moso bamboo seeds used in our experiments were collected at mid-august in 2016, from Dajing County, Guiling (E 110°17′-110°47′; N 25°04′-25°48′), Guangxi Zhuang Autonomous Region, China (Cheng et al., 2017). They were germinated on moist germination paper in culture dishes in the dark at room temperature (25°C) as described before (Chen D. et al., 2017), after which the seedlings were transferred to a greenhouse at 23/18°C under a 16/8 h light/dark cycle. They were planted in vermiculite and watered with 1/2 strength Hoagland’s nutrient medium once a week. Until they reached 2-month-old of age, the seedlings were subjected to abiotic stresses treatments and used to assay *MYB* gene expressions. For the low temperature stress, the plants were transferred to a growth chamber at 4°C under the same light and photoperiodic conditions. For salinity and drought stress, the seedlings were watered with 1/2 Hoagland’s solution with 200 mM NaCl or 20% polyethylene glycol (PEG) 6000 for 10 days, respectively. The control group and samples at low temperatures were also treated with standard 1/2 Hoagland’s solution simultaneously. The second or third leaves below the growth points were collected along a continual time course of 0, 1, 3, 6, 12, 24, 48 h, 4, 6, 8, and 10 days with three biological replicates for RNA preparation. We also selected some bamboo seedlings to be subjected to hormone treatments, including 200 mM MeJA (methyl Jasmonate), ABA and SA (salicylic acid). The leaves were harvested at 0, 1, 3, and 9 h. All samples were immediately frozen in liquid nitrogen and stored at -80°C until further analysis.

For tissue-specific analysis, the Illumina RNA-Seq data was used for analyzing the expression pattern of the *PheR2R3MYBs* in the different flowering stages of moso bamboo (Gao et al., 2014). Four floral developmental stages were defined according on the floral organs anatomical structure (F1, F2, F3, and F4). At stage F1, the flora bud begins to form. At stage F2, the floral organs mature gradually but do not flower. At stage F3, the flowers are in full bloom. At the last stage (F4), the pistils and stamens wither, and the embryo forms.

### Measurement of the Maximum Quantum Efficiency of Photosystem (PS)II

The maximum quantum yield of PSII (*Fv/Fm*) was measured along with the stress treatments using the Handy-Pea fluorometer from Hansatech (King’s Lynn, England) to ascertain the background level of photosynthetic down-regulation by fast chlorophyll fluorescence (Srivastava et al., 1995). The purpose of this measurement was to assess the effects of abiotic stress on photosynthesis with an independent measurement carried out in a different set of seedlings. Five leaves form each treatment (4°C, 200 mM Nacl and 20% PEG6000) were selected and fixed in the clips of the Handy-Pea and incubated in darkness for 15 min. The *Fv/Fm* ratio was measured under a continuous time course (0, 0.5, 1, 3, 6,12, 24, 48 h, 6, 8, and 10 d) in triplicate. The *Fv/Fm* ratios of the non-treatment samples (control) were also measured.

### Gene Expression Analysis

The transcriptome data of PheMYBs in developing flowers and shoots had been previously generated and processed. The gene expression levels of *PheR2R3MYBs* were calculated as reads per kilobase of exon model per million mapped reads (RPKM) units that was available from the NIH Short Read Archive (SRA) database^16^ (Gao et al., 2014). The heatmap was pictured using R.

The expression profiles of the *PheR2R3MYBs* under different treatments were analyzed by real-time quantitative RT-PCR (qRT-PCR). Each reaction contained 0.4 μL (10 μM) of each primer, 1.5 μL (30 ng) cDNA, 7.7 μL H_2_O and 10 μL SYBR Green I Master Mix (Roche, Mannheim, Germany) according to the manufacturer’s instructions in a final volume of 20 μl. The qRT-PCR parameters were 95°C for 5 min; followed by 45 cycles of 95°C for 10 s, 60°C for 10 s, and 72°C for 20 s. The gene-specific primers were designed using Primer 3.0, and their specificity was assessed with the BLAST tool using information provided by the local CDS database downloaded from Moso Bamboo SMRT^17^ (Wang T. et al., 2017). The primers used for qRT-PCR are shown in **Supplementary Table S1**. The tonoplast intrinsic protein 41 gene (*TIP41*) (Fan et al., 2013; Qi et al., 2013; Wu et al., 2016; Liu et al., 2017) was used as reference genes (Agarwal et al., 2006).

To verify the cold tolerance of the *PheMYB4-1* transgenic *Arabidopsis* lines, the total RNA was extracted form leaves of 4-week-old plants of wild-type (WT) and *PheMYB4-1*-overexpressing (OE) lines at 0, 2, 4 days after treatment (DAT) of 4°C. The qRT-PCR reaction and program are same as described above. We selected seven known cold-related *Arabidopsis* genes to detect gene expressions under chilling condition, including *CBF1*, *CBF2*, *CBF3*, *COR15A*, *RD29A*, *COR47*, *KIN1* (Chen et al., 2013), and the β-tubulin was used as reference gene (Agarwal et al., 2006). The primers are also shown in **Supplementary Table S1**. All the qPCR assays were performed with three biological and four technical replicates, and the quantitative analysis used the 2^-ΔΔCT^ method. Finally, the statistical analyses were performed using SPSS 19.0 software.

### Co-expression Network and Protein Interactions of PheMYBs

The expression correlation of the PheMYBs was calculated by Pearson correlation coefficient (PCC) using gene expression values from the high-throughput transcriptome data and qPCR data in R. Expression correlation data were used for the correlation network, and co-expressed gene pairs were filtered with a PCC cut-off of 0.85 as previously described (Smita et al., 2015). The network was further visualized and analyzed using Cytoscape version 3.4.0 (Shannon et al., 2003).

For the protein interaction networks, the homolog MYB proteins in rice were constructed by STRING^18^ using an option value > 0.7. The homolog proteins of the determined interactive rice proteins were identified in moso bamboo by reciprocal best BLASTP analysis.

### Overexpression of *PheMYB4-1* in *Arabidopsis* and Stress Treatments

The full-length coding sequence of *PheMYB4-1* was cloned into the pCAMBIA 2300 vector under the control of the modified CaMV 35S promoter (Cui et al., 2013). The pCAMBIA 2300-*PheMYB4-1* vector was introduced into *Agrobacterium tumefaciens* strain GV3101 for *Arabidopsis* transformation in the Col-0 background by the floral dipping method (Clough and Bent, 1998). Putative transgenic plants were screened on 1/2 Murashige and Skoog (MS) solid media supplemented with 50 mg l^-1^ kanamycin. After 3 days of vernalization at 4°C, they were transferred and germinated in a light incubator at 23/18°C under a 16/8 h light/dark cycle. About a week later, the seedlings were transferred to soil in greenhouse with same growth condition. Kanamycin-resistant of the T_3_ generation plants were subjected to *PheMYB4-1* gene expression analysis and stress tolerance evaluation.

To observe the effects of NaCl or mannitol on seed germination, two independent *PheMYB4-1*-OE lines (T_3_) and WT *Arabidopsis* plants were tested according to method previously described method (Wang N. et al., 2016). WT and *PheMYB4-1* OE lines seeds (a minimum of 50 seeds for each genotype) were sown on 1/2 MS medium plates with 150 mM NaCl or 200 mM mannitol, stratified at 4°C for 3 days and then transferred to long-day growth conditions as described above. The radicle emergence and cotyledon greening rates were measured after 3 and 7 days, respectively.

For chilling stress, the 4-week-old plants of WT and *PheMYB4-1* OE *lines* were treated at 4°C for 4 days. The samples were harvested at 0, 2, and 4 DAT. All samples were immediately frozen in liquid nitrogen and stored at -80°C until further analysis. For freezing tolerance, the seedlings were exposed to -10°C overnight in the dark. The phenotype was analyzed after 5 days of recovery at normal growth conditions.

### Subcellular Localization and Transcriptional Activation Analysis

Subcellular localization of the PheMYB4-1 protein was examined using the tobacco leaf transient expression system (Sparkes and Al, 2006). Full-length *PheMYB4-1* cDNA with the *BamHI* and *SpeI* restriction sites was separately amplified using the corresponding primers: PheMYB4-1-forward 5′CGGGATCCATGGGGAGGGCTCCGTGCT3′ and PheMYB4-1-Reverse 5′GGACTAGTAATCTGCGGCAACTGTT GCACGTC3′. The products were ligated to the pCAMBIA2300-35S-eGFP vector, while the PheMYB4-1 located at the N-terminus and GFP at the C-terminus in resulting constructs. After a 3 days post infiltration period, the transfected leaves were examined for green fluorescence signal using an FluoView FV1000 confocal microscope equipped with a 483-nm argon laser (Leica Microsystems) as described (Chai et al., 2014).

For transcriptional activation analysis, full-length PheMYB4-1 cDNA was individually fused in frame with the GAL4 DNA-binding domain in pGBKT7 (Clontech). The lithium acetate method was used to transferred into *Saccharomyces cerevisiae* AH109. The transactivation activity of each protein was evaluated according to literature (Chai et al., 2014).

## Results

### Identification of the PheR2R3MYB Gene Family in Moso Bamboo

A total of 202 *PheMYB* genes that contained complete MYB domains were identified and analyzed in the *P. edulis* genome, however, based on the FLNC reads, three genes were identified as mis-annotated, including two *1R-MYBs* (*PH01000383G0320*, *PH01000428G0040*) and one *2R-MYB* (*PH01001565G0340*). Additionally, three other *2R-MYB* genes from different scaffolds (PH01000003, PH01000190, PH01002931) were also investigated as novel MYB genes, and renamed as *PheMYB2R-110*, *PheMYB2R-111*, and *PheMYB2R-112* in our study. Thus, a primary dataset of 114 R2R3MYB proteins (112 R2R3MYBs, one R1R2R3 MYB protein and one 4R-type protein) and 88 MYB-related proteins was finally obtained. The results were consistent with the Pfam and SMART outcomes. Some of the resulting sequences were named according to the corresponding relationship between rice and moso bamboo, while others were numbered in sequence when no ortholog genes were available or the orthologous were not named in rice. Moreover, the physiochemical properties of all the PheR2R3MYBs were also analyzed (**Supplementary Table S2**). The GRAVY scores of the 114 *PheR2R3MYB* genes were all negative, indicating their soluble nature (Katiyar et al., 2012).

### Phylogenetic Analysis of the R2R3MYB Gene Family

**FIGURE 1:**
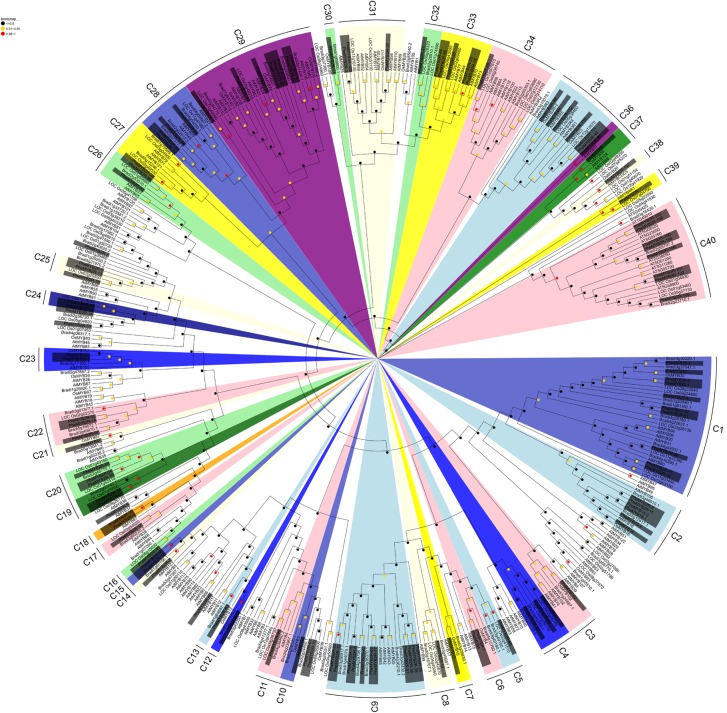
Phylogenetic analysis of the MYB transcription factors of moso bamboo, *Arabidopsis*, rice, and *Brachypodium*. Neighbor-joining phylogeny of 420 *R2R3MYB* genes of four species, as determined by MEGA 7. The colored shadow marks the subgroups of the MYBs. Numbers on branches are bootstrap proportions from 1000 replicates.

The protein sequences of 114 detected PheR2R3MYBs and all of the rice, *Brachypodium* and *Arabidopsis* R2R3MYBs were subjected to multiple sequence alignment. A phylogenetic tree was conducted in MEGA 7 using the NJ (**Figure 1**) and ML methods (**Supplementary Figure S1**), respectively. It seems that in ML tree, the PheR2R3MYB proteins were classified into more detail (69 clades). Although some minor modifications at interior branches existed, but for most proteins, they still have similar origin in both trees. As the phylogenetic trees derived from each method was quite similar, we took it as an indication of reliability of our clade designations. Here, only the NJ phylogenetic tree was further analyzed in our study. The sequence similarity and phylogenetic tree topology allowed us to classify the R2R3MYBs into 40 clades with at least 50% bootstrap support (**Figure 1**). A total of 11 subgroups were observed to share the MYBs among moso bamboo, rice, *Brachypodium* and *Arabidopsis*, suggesting the existence of a common ancestor between them. In contrast, 12 clades contained no *Arabidopsis* R2R3MYBs but only the members of the three grass-species, indicating some functional roles were either lost in *Arabidopsis* or acquired in the monocotyledonous lineages. We also observed species-specific clades that containing moso bamboo MYB proteins. For example, clade 15 and clade 18 both possessed a pair of *PheR2R3MYBs*. It was also easy to distinguish the putative orthologous genes of moso bamboo *R2R3MYBs* and rice/*Brachypodium*, since they were grouped into pairs within a clade, such as in clade 12, 13, 14, and 30 for *Bd-PheR2R3MYBs*, and clade 16, 21, 24, and 25 for *Os-PheR2R3MYBs*.

Peng et al. (2013a) previously showed that bamboo, *Brachypodium*, and rice from the BEP clade (Bambusoideae, Ehrhartoideae, and Pooideae) were more closely related than those belonging to the Panicoideae clade (maize, sorghum, and foxtail millet) (Peng et al., 2013a). In order to determine the evolutionary relationship of the *R2R3MYBs* from the BEP branch, we performed non-synonymous and synonymous substitution ratio (*Ka* and *Ks*) analysis of the duplicated genes. Six putative paralogous gene pairs in moso bamboo were identified using phylogeny-based and bidirectional best-hit methods in combination (Wu et al., 2016) (**Table 1**). Their *Ks* values ranged from 0.07 to 0.19, suggesting that the moso bamboo gene pairs were generated between ∼5 and 15 MYA. Moreover, the *Ka/Ks* ratios of all the *Phe-Phe* paralogous pairs were differed (**Table 1**), indicating that the PheR2R3MYB family had undergone a variety selective pressures during its history. We also identified three and four orthologous gene pairs of *Bd-Phe* and *Os-Phe* using the same methods, respectively (**Table 1**). The *Ks* values of *Bd-Phe* ranged from 0.6 to 0.92, suggesting that they were generated prior to ∼46 MYA. However, the *Ks* values of the five *Os-Phe* paris were from 0.34 and 1.32, indicating that they diverged prior to ∼26 MYA.

**Table 1:** Divergence between paralogous gene pairs and orthologous gene pairs.

Orthologous gene pairs		*Ks*	*Ka*	*Ka/Ks*	MYA
PheMYB88	OsMYB88	0.4394	0.3165	0.7203	33.80
PheMYB3R	LOC_Os07g04700	1.3212	0.9278	0.70224	102.00
PheMYB87	OsMYB87	0.6579	0.4374	0.664843	50.60
PheMYB2R-28	LOC_Os02g40530	0.4018	0.37	0.920856	30.90
PheMYB2R-60	LOC_Os02g41510	0.3396	0.2114	0.622497	26.10
PheMYB12	Bradi2g11676.2	0.9636	0.8935	0.927252	74.10
PheMYB2R-83	Bradi1g64687.1	0.8773	0.625	0.712413	67.50
PheMYB2R-2	Bradi1g60106.1	0.601	0.4901	0.815474	46.20
**Paralogous gene pairs**					
PheMYB2R-33	PheMYB2R-80	0.1201	0.0769	0.6403	9.24
PheMYB2R-56	PheMYB2R-68	0.1549	0.1991	1.285345	11.90
PheMYB2R-61	PheMYB2R-74	0.1119	0.0924	0.825737	8.61
PheMYB2R-54	PheMYB2R-39	0.169	0.132	0.781065	13.00
PheMYB2R-55	PheMYB2R-78	0.1941	0.2102	1.082947	14.90
PheMYB2R-4	PheMYB2R-10	0.0747	0.0809	1.082999	5.75


### Gene Structure and Protein Motif Analysis of the PheR2R3MYBs

**FIGURE 2:**
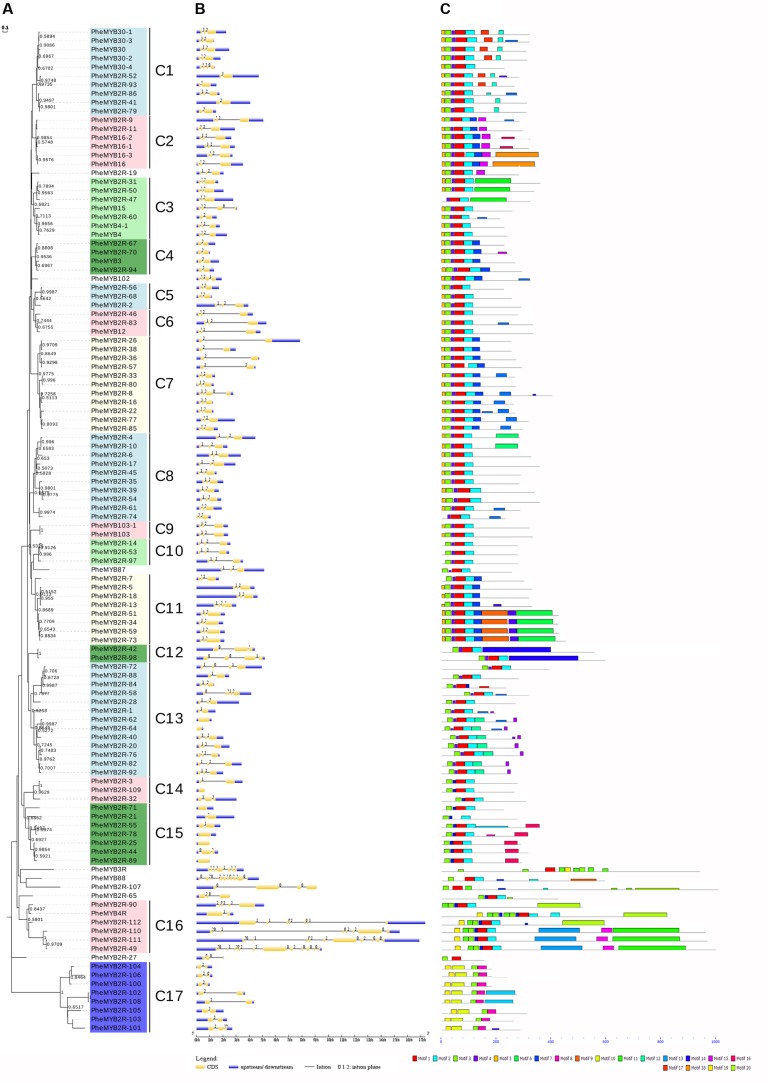
Phylogenetic relationships, gene structure and motif compositions of the moso bamboo R2R3MYB proteins. **(A)** The phylogenetic tree was constructed with MEGA 7 using the Neighbor–Joining (NJ) method with 1,000 bootstrap replicates based on a multiple alignment of amino acid sequences of R2R3MYB proteins from moso bamboo. Bootstrap values higher than 50% are shown on the nodes. The 17 major subfamilies are indicated with C1–C17 are marked with colorful backgrounds. **(B)** Exon/intron structures of *R2R3MYB* genes from moso bamboo. The exons and introns are represented by yellow boxes and black lines, respectively. The scale bar represents 1.0 kb. **(C)** Schematic diagram of the conserved motifs in the R2R3MYB proteins of moso bamboo, which were elucidated using MEME. Each motif is represented by a number in the colored box. The black lines represent the non-conserved sequences. The scale bar represents 200 aa.

To better examine the gene structure and conserved motifs among the moso bamboo MYBs, we constructed NJ and ML phylogenetic trees, respectively (**Figure 2A** and **Supplementary Figure S2**). As the results have a high degree of similarity (there are 14 clades in ML tree), we used the NJ tree for further analysis. The PheR2R3MYB proteins could be classified into 17 clades with at least 50% bootstrap support in NJ tree, and “the low bootstrap support for the internal nodes was in accordance with MYBs in other organisms” (Du et al., 2012a; He et al., 2016). The location of Clade 17 on the phylogenetic trees indicates its relatively ancient origin. Eight genes, including *PheMYB2R-19*, *PheMYB102*, *PheMYB87*, *PheMYB88*, *PheMYB3R*, *PheMYB2R-65*, *PheMYB2R-27, and PheMYB2R-107*, did not group with any other genes.

Most of the coding sequences of the *PheR2R3MYBs* were disrupted by introns (**Figure 2B**). In contrast, only 6% of the *R2R3MYBs* (seven sequences) did not possess introns. Most were typically spliced by two introns and three exons, while 25 *PheR2R3MYBs* possessed one intron and two exons, and the remaining genes possessed three to 12 introns. A total of six MYB sequences possessed more than six introns (*PheMYB2R-111*, *PheMYB88*, *PheMYB2R-110*, *PheMYB2R-112*, *PheMYB3R*, and *PheMYB2R-49*). Among these, *PheMYB88* was detected as homologous to *OsMYB88*, *AtMYB88*, and *AtMYB124* (Katiyar et al., 2012), while *PheMYB2R-49*, *PheMYB2R-110*, *PheMYB2R-111*, and *PheMYB2R-112* formed a distinct branch containing a complex exon/intron structure. Remarkably, genes in the same subfamily generally showed the same intron pattern and almost completely conserved intron positions. For example, the subfamily C2, C8, C9, C10, and C11 all lacked intron phase 0, while the subfamily C4 lacked intron phases 0 and 1 (**Figure 2B**). Generally, the splicing of each intron is occurred in one of three phases: phase 0, 1, or 2. In phase 0, the splicing occurs after the third nucleotide of the first codon; in phase 1, splicing occurs after the first nucleotide of the single codon; and in phase 2, splicing occurs after the second nucleotide (Du et al., 2012b). Here, the intron positions were further classified into 19 patterns (pattern a-s) according the splicing sites in R2 and R3 repeats of moso bamboo MYB proteins (**Supplementary Figure S3**). Approximately 56% of the moso bamboo genes exhibited pattern ‘n’, with conservative splicing sites of AG-L in the R2 repeat (intron 1) and NR-W in the R3 repeat (intron 2), as observed in other species (Du et al., 2012a,b).

Twenty conserved motifs in the R2R3MYB proteins of moso bamboo were identified using the MEME tool (**Figure 2C** and **Supplementary Table S3**). Most *PheR2R3MYBs* have motifs 1, 2, 3, 4, and 5, and motifs 1 and 3 represent the R2 and R3 MYB domains, respectively. As seen in **Figure 2**, most members of the same subgroup shared one or more identical motifs outside the MYB DBD, and closely related members were found to exhibit common motif compositions, suggesting functional similarities within the same subgroup. “The conserved intron patterns and motifs constitute independent criterions together for testing the reliability of our phylogenetic analysis” (Du et al., 2012b). Interestingly, the members of subfamily C17 possessed a distinct motif structure that has not before been reported in other species. A further analysis was also performed to investigate this phenomenon.

### Sequence Characteristics of MYB Proteins

**FIGURE 3:**
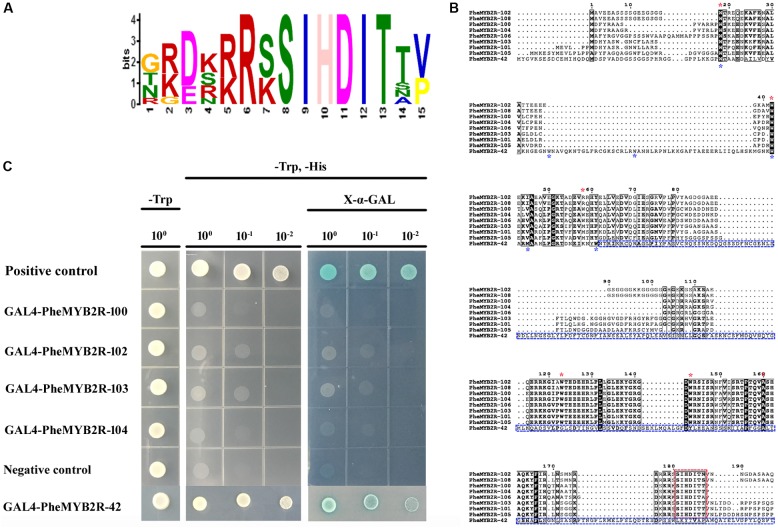
The investigation of sequence characteristic and transcriptional activation activities of eight PheMYB proteins from same clade. **(A)** Amino acid sequence context of a conserved motif-containing these eight proteins. The height of the letter representing an amino acid in each position reflects the difference in the frequency of its occurrence in the experimental sets. The core motif site is SIHDIT. **(B)** Amino acid sequence alignment between nine PheMYB proteins. The alignment was performed using ClustalX 2.1. A black background indicates conserved residues among all the proteins selected. The dashes indicate gaps introduced for better alignment. The positions of conserved W residues in R2 and R3 repeats in eight MYB proteins from Group 17 are indicated by red asterisks, and the third positions of R2 and R3 repeats in the MYBs from Group 17 were both substituted by other residues. The SIHDIT motifs in C terminal are marked with a red box. For PheMYB2R-42, the positions of W residues were marked by blue asterisks, and the C terminal following R3 repeat were indicated by blue box. No SIHDIT motif were detected in the PheMYB2R-42. **(C)** Full-length selected PheMYB proteins were tested for the transactivation activity. The empty pGBKT7 vector was used as a negative control, while the pGBKT7-53 and pGADT7-T were used as positive control. Survival of yeast cells on the selective media needs the presence of functional activator peptides. X-a-Gal was used to test the expression of a-galactosidase. The indicated concentration shows the dilution of yeast cell culture.

The sequence logos were produced using the deduced amino acid sequences of the R2 and R3 repeats, respectively (**Supplementary Figure S4**). Each repeat of the PheR2R3MYB family contained the highly conserved tryptophan (Trp W) residues, “indicating the indispensable role of these residues in maintaining the helix-turn-helix structure and serving as landmarks of plant MYB proteins” (Wang et al., 2015). However, in the eight proteins from clade 17, the conservative W residues were substituted by (Met, M)/(Arg, R) in W^74^ and alanine (Ala, A) in W^135^ at the conservative positions in the R2 and R3 repeats, respectively (**Figure 3B**).

Additionally, they all possessed a conserved motif of SIHDIT following the R3 domain, which has not previously been detected in other proteins (**Figure 3A**). We speculated whether the motif of SIHDIT affect the transcriptional activation activities in these MYB proteins. In a preliminary investigation of this, four genes in clade 17 were ligated into the pGBKT7 vector and introduced into the AH109 yeast strain (**Figure 3C**). The pGBKT7-53 and pGADT7-T and the empty pGBKT7 vector were used as the positive controls and negative control, respectively. The baseline activity of PheMYB2R-42 without SIHDIT motif in the C terminal was also established (**Figure 3B**). As a result, the PheMYB2R-42 showed a same growth pattern as positive control (**Figure 3C**), but the four PheMYBs from clade 17 displayed no transcriptional activation activities in the yeast system.

### Expression Profiles of PheR2R3MYB Genes During Flower Development

**FIGURE 4:**
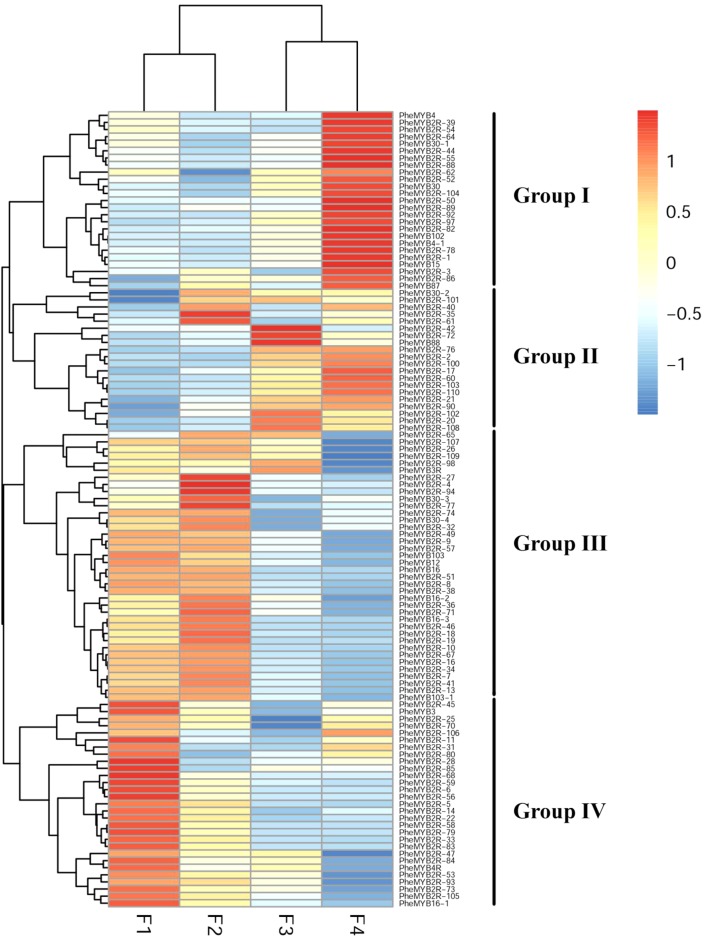
Expression profiles of moso bamboo *MYB* genes across development stages of moso bamboo flowers. Expression was visualized using heat maps. Blocks with colors indicate decreased (blue) or increased (red) expression levels.

MYB proteins are known to be associated with different aspects of plant development, and some of them have been identified as floral developmental regulators (Albert et al., 2011; Moreau et al., 2016). As described previously, most *PheMYBs* are upregulated during moso bamboo flower development, especially the genes involved in GA pathways (Gao et al., 2014). In this context, we investigated the expression patterns of this important gene family at different flower developmental stages using the published transcriptome data (**Supplementary Table S4**; Gao et al., 2014). We also selected six genes randomly to verify transcripts levels using qPCR, as previous RNA-seq data indicate that these genes exhibit similar expression patterns (**Supplementary Figure S5**). As observed in **Figure 4**, the *R2R3MYBs* could be classified into four groups (I, II, III, and IV) according to their expression profiles (Gao et al., 2014). Most *MYB* genes were highly expressed (RPKM > 10) in at least one stage during development, while only 12% of *PheR2R3MYBs* were lowly expressed (RPKM < 1) in more than two periods. Group I contained 24 members, and most were highly expressed during the last stage (RPKM > 100). Their expression levels were at least > 2 times higher than those in the other periods. In contrast, most members from Group III and IV were lowly expressed during the last stage. Instead, they were highly expressed at the F1 and F2 periods and decreased with development. Group II comprised 20 members, that were lowly expressed in the F1 stage, but the expressions increased steadily with flowering. We discovered some *R2R3MYB* genes to be highly expressed at specific periods. For example, *PheMYB2R-100*, *PheMYB4*, *and PheMYB4-1* were significantly upregulated at the F3 and F4 stages, while *PheMYB2R-7*, *PheMYB2R-34* were overexpressed in the earlier stages (RPKM > 100). This indicates their specialized regulatory role in flower development in moso bamboo.

### Expression Profiles of PheR2R3MYB Genes in Response to Different Stress Treatments

**FIGURE 5:**
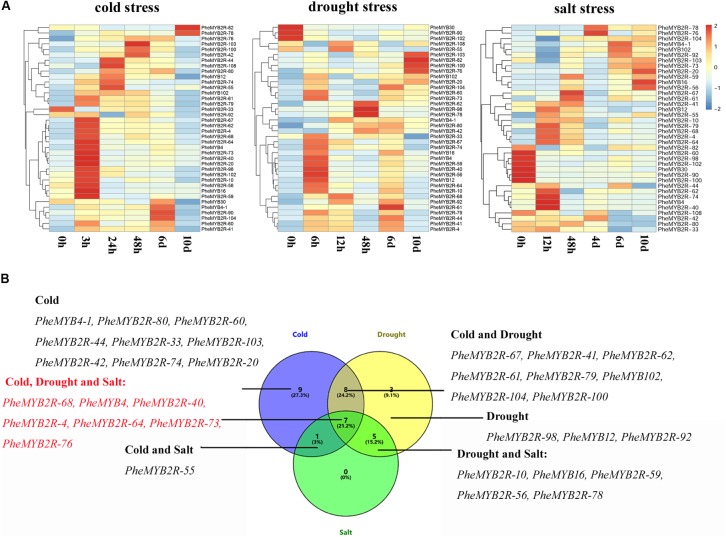
Expression patterns of selected *PheR2R3MYB* genes under various abiotic stresses and response model of moso bamboo. **(A)** The expression of *PheMYBs* after treated for 0, 3, 24, 48 h, 6 and 10 d under cold (4°C); 0, 6, 12, 48 h, 6 and 10 d under drought (20% PEG); 0, 12, 48 h, 4, 6, and 10 d under salt (200 mM NaCl). Expression was visualized using heat maps. Blocks with colors indicate decreased (blue) or increased (red) expression levels. **(B)** Venn diagram analysis of selected *PheMYB* genes was based on our qRT-PCR data.

As mentioned eralier, most MYB genes are regulated by stress and external factors. Here, *in silico* analyses also revealed that most *PheR2R3MYB* gene upstream sequences carry a variety of hormones and stress response elements (**Supplementary Figure S6**). To understand the transcriptional changes in *PheR2R3MYB* genes induced by abiotic stress, the seedlings were subjected to cold, drought and salt stresses up to 10 days. A total of 38 genes, including 22 genes chosen from stress-related subgroups and eight parolougous gene pairs were screened using their expression profiles from the qPCR under different stress conditions (**Figure 5**). Along with the abiotic stress treatments, the *Fv/Fm* value, as a sensitive indicator of stress in plants (Makarova et al., 1998), was also measured and used as a standard. Five time points of significant derease (3, 24, 48 h, 6 and 10 days for the cold treatment; 6, 12, 48 h, 6 and 10 days for the drought treatment; 12,48 h, 4, 6, and 10 days for the salt treatment) were selected for detecting the gene expression levels affected by the various stress treatments, respectively (**Supplementary Figure S7**).

For the cold treatment, the expression levels of all *PheR2R3MYBs* except for *PheMYB2R-33* were upregulated significantly under at least one time-course. They could be clearly divided into groups according to their expression profiles, and most genes were highly expressed after 3 h of cold treatment. Themajority of those expressions were also induced by drought treatment, and most also responded early to water deficiency (6 h). However, the expression of *PheMYB30*, *PheMYB2R-90*, and *PheMYB2R-102* were repressed by the drought treatment. Comparatively, more *PheR2R3MYBs* were downregulated under salt stress, and in addition to the three members (*PheMYB30*, *PheMYB2R-90*, and *PheMYB2R-102*) mentioned above, *PheMYB2R-60*, *PheMYB2R-82*, *PheMYB2R-100*, and *PheMYB2R-108* were also downregulated. Finaly, seven *PheR2R3MYBs* (*PheMYB2R-68*, *PheMYB4*, *PheMYB2R-40*, *PheMYB2R-4*, *PheMYB2R-64*, *PheMYB2R-73*, *PheMYB2R-76*) were indentified to be induced significantly by cold, drought and salt stress (foldchange > 2), and 14 *PheR2R3MYBs* (*PheMYB2R-67*, *PheMYB2R-41*, *PheMYB2R-62*, *PheMYB2R-61*, *PheMYB2R-79*, *PheMYB102*, *PheMYB2R-104*, *PheMYB2R-100*, *PheMYB2R-10*, *PheMYB16*, *PheMYB2R-59*, *PheMYB2R-56*, *PheMYB2R-78*, and *PheMYB2R-59*) responded signifcantly to the two treatments significantly (foldchange > 2). This suggests that these genes might constitute nodes of covergence for different stress response pathways. The expression patterns of the duplicated genes were found to be divergent (**Supplementary Figure S8**). Among the eight PheR2R3MYBs in paralogous pairs, the members in two pairs (*PheMYB2R-4*/*PheMYB2R-10* and *PheMYB2R-41*/*PheMYB2R-79*) presented similar expression patterns across the different stress treatments, and the remaning gene pairs all exhibited significant differences under at least one stress condition. However, most genes in paroulagous pairs except for *PheMYB2R-41*/*PheMYB2R-79* and *PheMYB2R-61*/*PheMYB2R-74* showed similar expression patterns during flower development in moso bamboo.

### Expression Correlation and Interaction Networks

**FIGURE 6:**
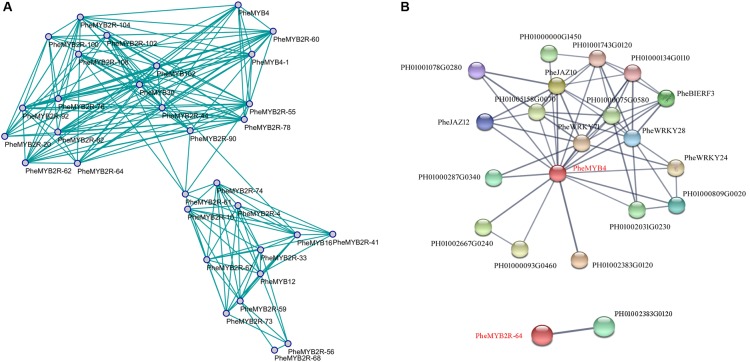
Co-expression network and interaction network of selected *PheR2R3MYB* genes in moso bamboo. **(A)** The model was built based on RNA-seq in flower development and our qPCR data in response to different stress treatments. The positive correlation value is shown by green color edges. **(B)** Interaction network of PheMYB4 and PheMYB2R-64 in moso bamboo. Colored balls (protein nodes) in the network were used as a visual aid to indicate different input proteins and predicted interactors. Protein nodes which are enlarged indicate the availability of 3D protein structure information. Gray lines connect proteins which are associated by recurring textmining evidence.

The co-expression network was constructed by connecting genes that are believed to be strongly co-expressed genes (PPC > 0.85) in both flower development and stress resistance (Smita et al., 2015). We discovered that some homologous genes were specific induced regardless of a stage of flower development or a stress resistance (**Figure 6A**). For example, *PheMYB4*, *PheMYB4-1*, and *PheMYB2R-60* all highly expressed in F4 stage and significantly induced by cold stress; *PheMYB2R-55* and *PheMYB2R-78* were salinity-induced and were all highly expressed in the F3 and F4 periods; *PheMYB2R-59* and *PheMYB2R-73* were highly expressed in F1 stage and upregulated by drought stress. Importantly, “genes with a high degree of connectivity either positive correlations were defined as hub genes” (Smita et al., 2015). In this study, we defined “hubs” as nodes with more than 12 connectivity in the whole network. Additionally, “candidate hub nodes that were significantly enriched in higher biological process levels were adopted as factors for potential hub genes in the network” (Smita et al., 2015). Among the 15 hubs, only *PheMYB4* and *PheMYB2R-64* were significantly enriched in response to different stress treatments (cold, drought, and salt) as well as being involved in the flower development process (**Figures 4**, **5**). To identify the PheMYB4- or PheMYB2R-64-associated proteins and protein complexes, prediction networks were constructed using STRING based on the interaction network of rice orthologous genes (**Figure 6B**). Finally, eighteen identified moso bamboo proteins were predicted to participate in the interaction network with PheMYB4 (interaction score > 0.7), but PheMYB2R-64 only interacted with one putative pre-mirna-splicing factor. In the PheMYB4-associated network, it interacted directly with 13 identified proteins. Among these, the WRKY proteins (PheWRKY24 and PheWRKY28), repressors of MeJA responses (PheJAZ10 and PheJAZ12) and ethylene-responsive TF (PheBIERF3) might be involved in biotic and abiotic stress resistance (Cao et al., 2005; Zhang et al., 2009; Lin, 2011; Li, 2012; Chujo et al., 2013), further indicating a necessary role for PheMYB4 in the response to stress signaling in moso bamboo.

### Functionality of the *PheMYB4-1* Gene in Transgenic *Arabidopsis*

**FIGURE 7:**
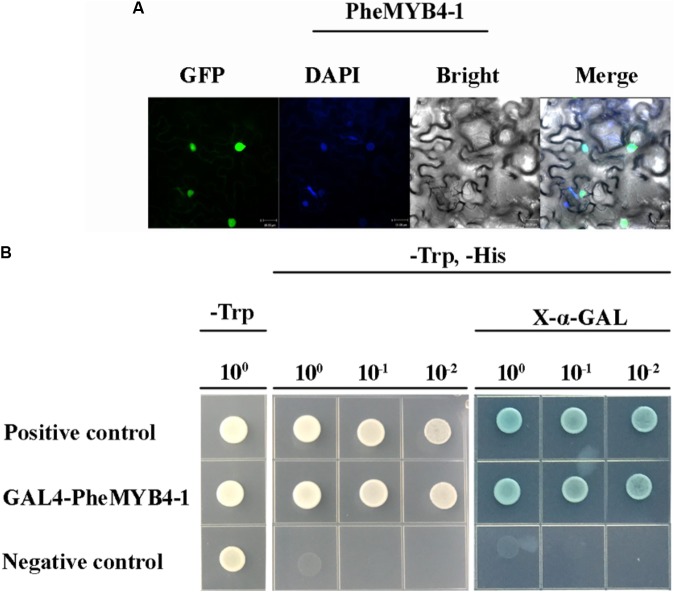
Subcellular localization, transcriptional activation analysis of PheMYB4-1 protein and phenotype analysis of the *PheMYB4-1* transgenic lines. **(A)** Confocal images of subcellular localizations of the *PheMYB4-1* in tobacco leaf epidermal cells; expression of the *PheMYB4-1*-GFP fusion gene was examined after 3 days by fluorescence and light microscopy; DAPI is used to stain the cell nucleus; bar = 10 μm. **(B)** Transcriptional activation analysis of the PheMYB4-1 fused with the GAL4 DNA binding domain in yeast, which was able to activate the expression of *His2* and *LacZ* reporter genes.

In this context, *PheMYB4-1* was identified as a homologous gene of *PheMYB4* based on its high shared sequence similarity (**Supplementary Figure S9B**). They all belong to the *OsMYB4* subfamily, and grouped with *OsMYB4*, *TaMYB2* and *Bradi5g15760.1* (**Supplementary Figure S9A**). As seen in **Figure 6**, *PheMYB4* and *PheMYB4-1* were all associated with the abiotic stress response of moso bamboo, displayed different expression patterns. We further analyzed the expression profiles of these two genes under stress hormone treatments; however, they exhibited completely opposite under ABA as well as MeJA treatments and all depressed by SA treatment (**Supplementary Figure S9C**). This indicated that *PheMYB4-1* might be involved in a different stress signaling pathway comparing with *PheMYB4*. We further amplified the coding region of *PheMYB4-1* and fused it to the N-terminal of eGFP and pGBKT7 vector, respectively. The subcellular localization analysis indicated that the *PheMYB4-1* was localized in the nucleus and showed strong transcriptional activity in the yeast system, likely functioning as a TF (**Figures 7A,B**). *PheMYB4-1* was further ectopically expressed in *Arabidopsis*, and 12 homozygous T_3_ lines were obtained. Here, we selected three lines with different classes of gene expression (**Supplementary Figure S10A**): low expression (LE), line 4-1-8; high expression (HE), line 4-1-9 (HE1) and line 4-1-4 (HE2). The HE plants were characterized by larger leaves in a vegetative growth period, which is depending on the *PheMYB4-1* expression level, but *PheMYB4-1* overexpression did not affect the number of rosette leaf prior to bolting (**Supplementary Figure S10B** and **Supplementary Table S5**). Moreover, the structurally defective in *PheMYB4-1* OE seeds was analyzed by mean of scanning optical microscopy (**Supplementary Figure S10C**). Comparing with WT, the seeds of *PheMYB4-1* OE lines displayed different degrees of malformation (**Supplementary Table S5**). In HE-2, some seeds had lost their embryos completely (**Supplementary Figure S10D**), which may affect the rate of seed germination. So, we mainly used the HE-1 and LE lines in the following experiment.

**FIGURE 8:**
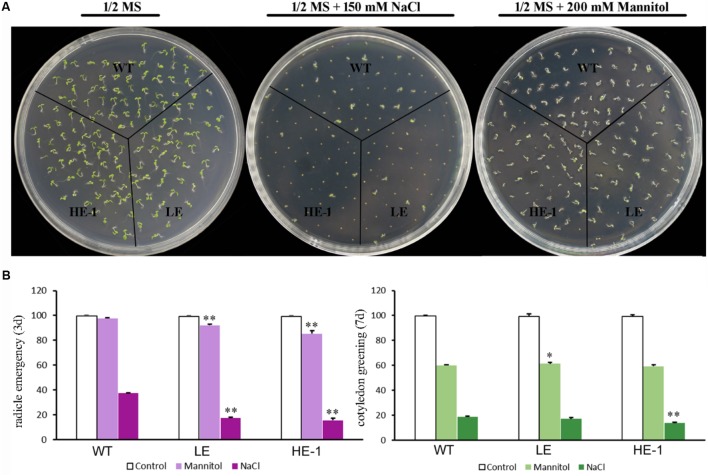
Seed germination of wild-type (WT) and *PheMYB4-1*-overexpressing *Arabidopsis* under mannitol or NaCl stress. **(A)** Seeds were germinated on 1/2 MS agar plates with or without various concentrations of mannitol or NaCl. **(B)** Germination percentages were determined in terms of radical emergence after 3 days and cotyledon greening after 7 days. Photographs were taken 3 days after mannitol or NaCl treatment. Data are reported as the average value of three replicates using > 50 seeds for each genotype. The experiments were repeated at least three times with similar results, and data from one representative experiment are presented. Different number of the asterisk represents the statistically different ^∗^*P* < 0.05; ^∗∗^*P* < 0.01.

The germination rates and cotyledon greening of WT and *PheMYB4-1*-OE *Arabidopsis* seeds were compared under the presence or absence of mannitol or NaCl (**Figures 8A,B**). The results showed that 3 days after germination, the radicle emergence rates of WT seeds decreased from 100% (1/2 MS) to 96% (200 mM mannitol). The *PheMYB4-1*-OE plants displayed a comparatively sensitive phenotype under mannitol stress. Approximately 84 and 68% of *PheMYB4-1*-OE seeds could germinate in the presence of 200 mM mannitol. Germination tests were also performed in the presence of 150 mM NaCl. Apparently, the germination rates of the *PheMYB4-1*-OE seedlings were decreased to 6 and 4% were more seriously impacted than in the WT seedlings (16%). As respect, the cotyledon greening of *PheMYB4-1*-OE lines was also decreased than WT seedlings under both drought and salt treatments, but the difference is not so obvious. These results provide evidence that the overexpression of *PheMYB4-1* have been slightly enhanced sensitivity to mannitol and NaCl of the transgenic lines during the germination stage.

**FIGURE 9:**
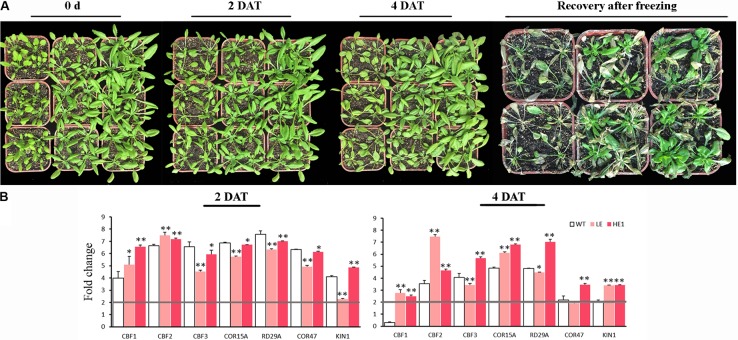
Increased cold and freezing tolerance of the *PheMYB4-1* overexpression lines. **(A)** Chilling and freezing test carried out using wildtype, and *PheMYB4-1* overexpression lines (LE and HE-1) plants. All plants were grown in soil at 22°C for 3 weeks and then transferred into a chamber at 4°C and –10°C for 4 days and 8 h, respectively. The plants were photographed at 0, 2, and 4 days after cold stress and 5 days recovery after freezing stress, respectively. **(B)** Relative mRNA levels of cold responsive genes examined by real-time quantitative PCR. Expression levels of *CBF1*, *CBF2*, and *CBF3* were increased to a much greater extent in LE and HE lines than in wild-type lines under cold treatment. The expression levels of the downstream genes such as *KIN1*, *COR15A*, *COR47*, and *RD29A* in HE lines were also elevated to a much higher level than those in wild-type or LE lines under low temperature. The expressions of the cold-related genes were compared with the control in 0 h. The different color boxes correspond to log2 transformed value. Different number of the asterisk represents the statistically different ^∗^*P* < 0.05; ^∗∗^*P* < 0.01.

Cold and freezing stress damage on *PheMYB4-1*-OE plants was evaluated (**Figure 9**). Under chilling condition, the leaves of WT seedlings began to drop at 2 DAT, however, the LE and HE lines did not show the dwarf phenotype evidently. At 4 DAT, the leaves of LE and HE lines were slouched, but they still remained green and alive compared with WT plants. At freezing treatment, all the plants were frostbite in different degrees. After 5 days recovery, the WT seedlings were almost wilting and died, but the LE and HE lines were still kept in green and rapidly restored growth (**Figure 9A**). To determine the molecular mechanisms by which *PheMYB4-1* regulates cold tolerance, we examined changes in expression of *CBF* genes, which are known regulators of cold response (**Figure 9B**). At early stages (2 DAT), the cold related genes were all upregulated significantly (foldchange > 2) in all samples. Among these, *CBF1* and *CBF2* were both significantly induced in the LE and HE1 lines than the WT seedlings, but the expression level of *CBF3* and *CBF* downstream genes (*COR15A*, *RD29A*, *COR47*) in *PheMYB4-1* OE lines were lower than WTs. After 4 days treatment, consistent with the changes in *CBF* genes, the expression levels *KIN1*, *COR15A*, *RD29A* and *COR47* in *PheMYB4-1* OE plants were elevated to levels higher than those in WT lines especially in the HE lines. These results suggest that the increased cold and freezing tolerance in *PheMYB4-1* OE plants resulted from the much higher expression of *CBF* genes and their downstream genes under cold treatment.

## Discussion

### R2R3MYBs Gene Family in Moso Bamboo

The R2R3MYB gene family is one of the largest families of TFs in plants. In a genome-wide and FLNC database screen, 202 *MYB* genes that harbored conserved domains in moso bamboo were identified, including 114 R2R3MYBs and 88 *MYB*-related genes. Different numbers of *R2R3MYB* genes exist in *Arabidopsis* (135), *Brachypodium* (81), rice (90), wheat (28), barely (61), and maize (119). Of these plants, the genome size of moso bamboo is comparable to maize (2,021 and 2,300 Mb), but larger than *Brachypodium* (300 Mb), *Arabidopsis* (164 Mb), and rice (441 Mb) (Burr, 2002; Schulman, 2009; Peng et al., 2013a; Wang et al., 2014). Barley and wheat (Triticeae) possess larger genomes, >5 Gb and 17 Gb (Schulte et al., 2009; International Wheat Genome Sequencing Consortium, 2014), but contain 61 and 28 R2R3MYB proteins, respectively (Zhang et al., 2012; Tombuloglu et al., 2013). In this context, the number of R2R3MYBs in moso bamboo is larger than that in *Brachypodium*, rice, wheat, and barley but less than that in *Arabidopsis*, indicating that the abundance of *MYB* genes in a species may be related to genome duplications (segmental/tandem), rather than the genome size. To date, the genome assembly of moso bamboo is still in draft, covering ∼95% of the whole genomic region (Zhao et al., 2014). Fortunately, the FLNC reads were significantly improved using the current annotations (Wang T. et al., 2017), which helped us to eliminate erroneous genes and identify missing genes.

Phylogenetic analysis of R2R3MYB proteins in *Arabidopsis*, *Brachypodium*, rice, and moso bamboo (**Figure 1**) indicated a close relationship between the *Brachypodium*, rice, and moso bamboo R2R3MYB proteins from the BEP clade (Peng et al., 2013a). Moso bamboo underwent a WGD event 7–12 MYA following a few tandem duplicates, which in turn has generated gene duplication and gene loss events (Peng et al., 2013a). In order to assess the occurrence of the WGD event in the MYB gene family in moso bamboo, we estimated the *Ks* and *Ka* models of paralogous and orthologous genes and the T value (T = Ks/2λ, λ = 6.5 × 10^-9^) for each gene pair (Wu et al., 2016; Liu et al., 2017). We discovered that all moso bamboo paralogs were generated around the WGD event. Peng et al. (2013a) estimated that the divergence time for moso bamboo and rice was 48.6 MYA, while that for moso bamboo and *Brachypodium* was 46.9 MYA. The orthologs of most *Phe-Os* and *Phe-Bd* all underwent gene evolution close to their divergence time, but *PheMYB3R/LOC_Os07g04700* was generated approximately 102 MYA, which is far earlier than the divergenece time for moso bamboo and rice. In general, *Ka/Ks* ratios less than 1, equal to 1, and greater than 1 indicate negative or stabilizing selection, neutral selection, and positive selection, respectively (Cannon et al., 2004). In our study, the paralogous gene pairs of moso bamboo underwent a variety of evolutionary selective pressures, with *Ka/Ks* ratios ranging from 0.64 to 1.28, indicating that the selective constraints have been unstable in the evolution of *MYB* genes in moso bamboo.

### Sequence Conservation and Characteristics of PheR2R3MYBs

Protein conservation in the *PheMYB* gene family was studied. It is understood that the highly conserved Trp amino acid residues (W) distributed in the third helix are important for DNA-binding activity of MYB proteins (Ogata et al., 1994). Thus, “the highly conserved characteristics of the third helix may indicate functional conservation among different plant species during MYB evolution, while species-specific genes may be derived from key residue exchanges in this region” (Du et al., 2012a). Interestingly, the members from Group 17 were characterized by a special base substitution at W^74^ and W^135^ by M and A, respectively. The base substitution may seriously impair DNA-binding activities, but also possibly result in the recognition of novel target genes. Therefore, it is necessary to verify the DNA-binding capability of these unique MYBs using EMSA or other experimental approach.

Additionally, these proteins all possess a specific motif in the C-terminal that distinguishes them from other subfamilies. In order to verify whether this element has an effect on the transcriptional activity of genes. We obtained full-length cDNA sequences of *PheMYB2R-100*, *PheMYB2R-102, PheMYB2R-103*, and *PheMYB2R-104* and observed their transcriptional activity by fusing them in-frame with the GAL4 DNA-binding domain in pGBKT7 and transferring the vector into *S. cerevisiae* AH109. The PheMYB2R-42 without SIHDIT motif was also selected randomly to further verify the prediction. However, on the contrary with the PheMYB2R-42 and the positive control, the genes from Group 17 did not activate the His and LacZ reporter genes. It indicates the genes from Group 17 might have transcriptional repression activity because of the specific motif SIHDIT, but the exact role of motif SIHDIT needs more study. In the other side, the members in this group might have lost their transcriptional activation regions during evolution. Above all, an intensive analysis is required to test the functional roles of these characterized *MYB* genes in moso bamboo.

### PheMYB Proteins Are Involved in Flower Development and the Stress Response of Moso Bamboo

Over the past few decades, extensive studies have been conducted into the roles of MYB proteins in regulating important processes in plants, including development, metabolism, and the environmental stress response (Ambawat et al., 2013; Li et al., 2015). Some *MYB* genes were found to be floral developmental regulators, including *AtMYB21*, *24*, and *103* in *A. thaliana* (Shin et al., 2002; Yang et al., 2007; Zhang et al., 2007). In apple, a stamen-specific MYB39-like TF (*MdMYB39L*) was involved in stamen development and pollen tube growth as a target of sorbitol. Suppressing *MdMYB39L* expression in the pollen significantly reduced the expression of its putative target genes and pollen tube growth (Meng et al., 2017). As described previously, MYB TFs were identified in the panicles of moso bamboo (Peng et al., 2013a) and may be differentially expressed during flower development (Gao et al., 2014). In this context, most *PheMYB*s, such as *PheMYB103* and *MYB103-1*, showed high expression levels during the floral bud formation and gradual organ maturation stages (F1 and F2). In rice, “the *OsMYB103* RNA transcript was most abundantly expressed in the flowers, specifically in the tapetum, premeiotic pollen mother cells, and meiotic PMCs. *OsMYB103*-RNAi transgenic lines grew normally during their vegetative phase but displayed reduced male fertility; a phenotype that was associated with reduction of *OsMYB103* transcript levels” (Zhang et al., 2010). This suggests regulatory roles for *PheMYB103* and *MYB103-1* in floral meristem formation, particularly in the anther development, in moso bamboo. *PheMYB2R-42* and *PheMYB2R-98* were highly expressed in the full-blossoming stage (F3). They were found to be the orthologous genes of *OsGAMYB* (*Loc_Os01g59660*) in our study. In rice, “*OsGAMYB* is important for floral organ development and essential for pollen development.” In the reproductive phase, “shortened internodes and defects in floral organ development, especially defects in pollen development, were observed in the *osgamyb* mutants” (Kaneko et al., 2004). Therefore, *PheMYB2R-42* and *PheMYB2R-98* might also be related to pollen development in moso bamboo. Moreover, they may act as the targets of miR159 like in other species and participate in other tissue developmental processes (Alonso-Peral et al., 2010; Akkaya et al., 2011; Csukasi et al., 2012; Chin et al., 2016; da Silva et al., 2017; Wang M. et al., 2017).

“Environmental conditions, such as salinity, moisture, and temperature, affect plant development, growth, and productivity” (Li et al., 2015). Therefore, responses and adaptations to abiotic stresses are vital mechanisms for plant survival. Increasing amounts of research has indicated that MYB proteins play fundamental roles in the regulation of gene expression in response to environmental change, where they act as active players in abiotic stress signaling (Ambawat et al., 2013; Li et al., 2015). In moso bamboo, the overexpression of *PeMYB2* (named *PheMYB2R-73* in our study) could strengthen the tolerance of the transgenic Arabidopsis lines under salt stress conditions (Xiao et al., 2013). Moreover, “the MYB TFs might play crucial roles in regulating, acclimating, and modulating gene expression in photosynthetic processes in response to the high-light stress of moso bamboo” (Zhao et al., 2016). Discovery of regulatory *cis*-elements in the promoter regions is essential for understanding the potential roles of *MYB* genes. The majority of stress- and hormone- related elements were randomly distributed in the upstream sequences of *R2R3MYB*s, implying that most *PheMYB* genes are involved in the abiotic stress response. However, *in silico* analyses may present some issues. Here, we first analyzed the effects of cold, drought, and salt stress on the expression patterns of *PheMYBs*. The majority of selected *PheMYBs* were involved in the cold and drought response (as observed in other species; (Katiyar et al., 2012; Smita et al., 2015), and a total of seven genes responded significantly to all the stress treatments (fold changes > 2). Among these, *PheMYB2R-4* and *PheMYB2R-68* were specific to moso bamboo and may constitute novel stress regulators in the evolutionary history of moso bamboo. *PheMYB2R-40*, *PheMYB2R-76*, and *PheMYB2R-64* clustered with *OsMYB2* and the members from subgroup 20 in *Arabidopsis* (Dubos et al., 2010; Yang et al., 2012). In the orthologous genes, “*AtMYB2* controls the ABA induction of salt and dehydration responsive genes,” functions as a direct transcriptional activator for miR399, and may be involved in the phosphate starvation response (Urao et al., 1996; Baek et al., 2013). *AtMYB108* is known to respond to both biotic and abiotic stress conditions (Mengiste et al., 2003). In rice, *OsMYB2* is involved in salt, cold, and dehydration tolerance, and *OsMYB2*-overexpression plants were more sensitive to ABA than WT plants (Yang et al., 2012). These findings suggest crucial roles of *PheMYB2R-40*, *PheMYB2R-76*, and *PheMYB2R-64* in enhancing tolerance in response to multiple stress conditions in moso bamboo.

### Hub Gene PheMYB4 and Its Homolog PheMYB4-1 Exhibit Biological Significance

We used co-expression network-based expression analysis in both flower development and stress resistance to dissect the MYB transcriptional regulatory networks and their correlations in moso bamboo. Based on their degree of connectivity, the hub genes *PheMYB2R-64* and *PheMYB4* were identified to play a regulatory role and correlated with other *PheMYBs* in the complex feedback network. Finally, we discovered that the PheMYB4 protein has a vital functional role in the complex regulatory network associated with environmental stress, including ABA, MeJA, cold, drought, and salinity treatments, as well as the flower development process. In rice, “*OsMYB4* was demonstrated to control a hierarchical network comprised of several regulatory sub-clusters associated with cellular defense and rescue, metabolism and development. It regulates target genes either directly or indirectly through the intermediary MYB, ERF, bZIP, NAC, ARF and CCAAT-HAP TFs” (Park et al., 2010). Additionally, the overexpression of the *OsMYB4* gene in Arabidopsis increases tolerance to abiotic, environmental and biotic stresses (Vannini et al., 2004, 2006; Mattana et al., 2005; Pasquali et al., 2008). It significantly contributes to the tolerance of rice cultivars to sheath blight and increases the production of specific bioactive hydroxycinnamates of transgenic tobacco and *Salvia sclarea* plants (Docimo et al., 2012; Singh and Subramanian, 2017). In this context, the seeds development of *PheMYB4-1*, a homologous gene of *PheMYB4*, overexpressing transgenic lines were significantly affected depending on overexpression level. We also found the *PheMYB4* and *PheMYB4-1* displayed same expression pattern during flower development, which highly expressed at the immature embryo formation period. It suggests that this gene pair should both associate with embryo development in the moso bamboo seeds. Park et al. (2010) pointed out that “a supra-optimal expression of *OsMYB4* caused the misexpression of alternative targets with costly trade-offs to panicle development,” further influencing the grain yield by dramatically reducing the average seed set. Clearly, in rice, “the OsMyb4 network defines an intricate interplay of growth- and stress-related responses.” It indicates necessary roles of *PheMYB4* and *PheMYB4-1* in regulating plant growth and plant stress response of moso bamboo.

### *PheMYB4-1* Is Involved in an ABA-Independent Pathway in Response to Abiotic Stress

The *PheMYB4-1* and *PheMYB4* showed a high sequence similarity and belong to a highly conserved *OsMYB4* subfamily among monocots (Baldoni et al., 2013). Although they were all induced by cold condition significantly (**Supplementary Figure S11**), they showed different expression trend under salt and drought treatment. For drought, *PheMYB4* was continued significantly induced after 6 h treatment, but *PheMYB4-1* was upregulated after 12 h treatment. For salt, *PheMYB4* was significantly induced earlier (12 h after treatment) and then repressed, while *PheMYB4-1* was suppressed earlier and slightly up-regulated later (6 and 10 days). It indicates that the regulatory roles of *PheMYB4* and *PheMYB4-1* were similar in response to cold stress but different under salt or drought treatment. “A boost in ABA biosynthesis is a frequently observed plant response to abiotic stresses (And and Bartels, 1996), and this has the effect of triggering a number of ABA-dependent genes involved in the stress response” (Fujita et al., 2004; Li et al., 2010; Zhou et al., 2010, 2018; Wang et al., 2018). For most plants, they involved in a crosstalk of both ABA-dependent and ABA-independent pathways to better adapt to survival in environmental stresses (Riera et al., 2005; Barrero et al., 2006; Yoshida et al., 2014). The *PheMYB4* and *PheMYB4-1* appear completely opposite expression patterns in response to exogenous ABA, suggesting they should be involved in ABA-dependent and ABA-independent pathways in stress resistance of moso bamboo, respectively.

To further examine the role of PheMYB4-1 protein, we generated the transgenic *Arabidopsis* plants overexpressing *PheMYB4-1*. While on the contrary to the positive role of *OsMYB4* in participating in salt and drought defense (Vannini et al., 2006, 2007), the seed germinations of transgenic lines were both lower than WT seedlings under NaCl and mannitol treatments. It indicates the *PheMYB4-1* decrease the tolerance of drought and saline environment of transgenic *Arabidopsis*. However, the regulatory role was not in line with the gene expression of *PheMYB4-1* in moso bamboo (**Supplementary Figure S11**), especially in drought condition. Similar phenomenon was also observed. For example, “the *TaCHP*, a zinc finger protein gene was down-regulated by abscisic acid and salinity stress in wheat, but it plays a positive role in salt tolerance in transgenic *Arabidopsis*” (Li et al., 2010). “The expression of a novel CaWDP1 protein was induced by ABA, drought and NaCl treatments in *Capsicum annuum*, but *CaWDP1*-OE transgenic *Arabidopsis* plants exhibited a drought-susceptible phenotype” (Park et al., 2017). In this context, *PheMYB4-1* was identified to have transcriptional activation activity. Therefore, although the *PheMYB4-1* was induced by drought and salt stresses in moso bamboo, it possibly stimulates some drought or salt suppressors expressions to increase sensitivity to both water-deficit and salinity stress in moso bamboo. On the other side, the *PheMYB4-1* was significantly upregulated in later stages under drought and salt conditions (after 12 h drought treatment and 6 days salt treatment), suggesting a role as “late response factor.” It possibly be upregulated by other early drought or salt response factors as targets. Finally, “the effect of *Osmyb4* on stress tolerance likely depends on the genetic background of the transformed species” (Laura et al., 2010). Thus, we cannot exclude the possibility that the difference between expression pattern and the actual functionality in the heterologous transgenic system might due to the different genetic background of moso bamboo and *Arabidopsis*. Overall, the functions of *PheMYB4-1* under drought and salt still need more experimental verification.

The *PheMYB4-1* transgenic lines showed enhanced cold and freezing resistance than WT seedlings. Under low temperature condition, *CBF* genes were induced first (2 DAT) and other cold-related genes were significantly induced subsequently in *PheMYB4-1*-OE line after 4 days treatment. In *Arabidopsis*, the *RD29A*, *COR15A* both showed no changes in *AtMYB15* overexpression and knock-down lines (Agarwal et al., 2006), but the *CBF* and a serious of cold-related genes were rapidly and simultaneously upregulated in amiRNA14 *Arabidopsis* lines (Chen et al., 2013). These differences suggest that the functional mechanisms of *PheMYB4-1* and *AtMYB15* or *AtMYB14* in cold tolerance differ, but it should relate to ABA-independent cold-response pathway as *OsMYB4* (Vannini et al., 2004). We proposed that *PheMYB4-1* might bind to the *CBF* promoters to induce the *CBF* expression directly and the upregulated CBF genes further activate the downstream cold-related genes expression. The data reported here suggest that the *PheMYB4-1* is involved in response to low temperature. However, as the heterogenous transformation in *Arabidopsis* (dicot) cannot reacted the actual function intuitively in the phylogenetically distant of *P. edulis* (monocot), more experiments are still needed to verify its mechanism in cold environment condition of moso bamboo.

In summary, the *PheMYB4-1* was identified as a cold but not drought, salt and ABA-induced gene. It suggests that its function to be specifically related to the ABA-independent cold response (Agarwal et al., 2006). Moreover, like most other gene pairs analyzed in our study, the expression patterns of the *PheMYB4* and *PheMYB4-1* were differed regardless of the drought and salt stresses or stress hormone treatments, but they were in similar under cold treatment. This suggests that this gene pair should partially functional diverged in its plant stress responses during the evolutionary history of the MYB family (Peng et al., 2013a). Therefore, we proposed that the *PheMYB4* should be also involved in stress response and might enhanced a tolerance to environmental stress of moso bamboo.

## Author Contributions

JG contributed to the conceptual and experiment designs. DH conducted the experiments. ZC and LX performed the RNA-seq data analysis. XL contributed the reagents, materials, and analysis tools. JL and SM revised the manuscript. DH wrote the report. All the authors have commented, read, and approved the final manuscript.

## Conflict of Interest Statement

The authors declare that the research was conducted in the absence of any commercial or financial relationships that could be construed as a potential conflict of interest.

## Footnotes

1 https://www.ncbi.nlm.nih.gov/

2 http://202.127.18.221/bamboo/index.php

3 http://pfam.sanger.ac.uk/

4 http://www.ncbi.nlm.nih.gov/Structure/cdd/cdd.shtml

5 http://www.forestrylab.org/db/PhePacBio/phe/Jbnest.php

6 http://www.expasy.ch/tools/protparam.html

7 http://www.arabidopsis.org/

8 http://rice.plantbiology.msu.edu/

9 http://www.phytozome.net/

10 https://www.ebi.ac.uk/Tools/msa/clustalo/

11 https://www.ebi.ac.uk/Tools/msa/muscle/

12 http://gsds.cbi.pku.edu.cn/index.php

13 http://meme-suite.org/tools/meme

14 http://weblogo.berkeley.edu/logo.cgi

15 http://bioinformatics.psb.ugent.be/webtools/plantcare/html/

16 http://www.forestrylab.org/db/PhePacBio/

17 https://www.ncbi.nlm.nih.gov/sra/?term=SRR1187864

18 http://string-db.org/

## Supplementary Material

FIGURE S1 The maximum likelihood phylogeny tree of the MYB transcription factors of moso bamboo, *Arabidopsis*, rice, and *Brachypodium*. The colored shadow marks the subgroups of the MYBs. Numbers on branches are bootstrap proportions from 1000 replicates.

FIGURE S2 The maximum likelihood phylogeny tree of the MYB proteins in moso bamboo. Numbers of branches are bootstrap proportions from 1000 replicates.

FIGURE S3 Schematic of the intron distribution patterns within the moso bamboo R2R3MYB DNA-binding domains. Alignment of MYB domains is representative of 19 intron patterns, designated a to s. Locations of introns are indicated by black triangles, and the number above the black triangles indicates the splicing phases of the MYB domain sequences: 0 refers to phase 0; 1 to phase 1; and 2 to phase 2. The number of PheMYB proteins with each pattern is presented on the right.

FIGURE S4 **(A,B)** The logos of R2 and R3 MYB repeats across all the R2R3MYB proteins of moso bamboo.

FIGURE S5 Expression verification of *PheMYB* genes in different stages of developmental flowers (F1 to F4).

FIGURE S6 The statistics of total number of *PheMYB* genes including corresponding *cis*-acting elements (light gray dot) and total number of *cis*-acting elements in PheMYB gene family (black gray box). Based on the functional annotation, the *cis*-acting elements were classified into three major classes: stress-, hormone-, or development-related *cis*-acting elements. The annotation of the *cis*-elements: WUN-motif, wound responsive element; TC-rich repeats, involved in defense and stress responsiveness; MBS, MYB binding site involved in light responsiveness and drought-inducibility; LTR, involved in low-temperature responsiveness; HSE, *cis*-acting element involved in heat stress responsiveness; ARE, essential for the anaerobic induction; Box-W1, fungal elicitor responsive element; GC-motif, enhancer-like element involved in anoxic specific inducibility; MEJA, involved in the MeJA-responsiveness; EIRE: elicitor-responsive element; TCA-element, involved in salicylic acid TCA-element, involved in salicylic acid; TGA-element/TGA-box/AuxRE/AuxRR-core: part of an auxin-responsive element; GARE-motif/ P-box/TATA-box: *cis*-acting element involved in gibberellin-responsiveness; ABRE/Motif IIb/CE1/CE3: abscisic acid responsive element; ERE, ethylene responsive element; HD-Zip1, differentiation of the palisade mesophyll cells; HD-Zip2, control of leaf morphology development; RY-element, seed-specific regulation; MBSI, MYB binding site involved in flavonoid biosynthetic genes regulation. Skn-1 motif and GCN4_motif, *cis*-regulatory element involved in endosperm expression; CCGTCC-box, related to meristem specific activation; CAT-box: *cis*-acting regulatory element related to meristem expression; circadian: *cis*-acting regulatory element involved in circadian control; As-2-box: involved in shoot-specific expression and light responsiveness.

FIGURE S7 Effects of abiotic stresses (cold, drought and salinity) on chlorophyll fluorescence parameters of moso bamboo leaves. The *Fv/Fm* values of moso bamboo leaves under **(A)** control, **(B)** cold stress, **(C)** drought stress, and **(D)** salt stress. Different number of the asterisk represents the statistically different ^∗^*P* < 0.05; ^∗∗^*P* < 0.01. They began to significantly decrease after 3 h cold treatment, 6 h drought treatment and 12 h salt treatment, respectively.

FIGURE S8 The expression patterns of eight selected gene pairs under different stress treatments.

FIGURE S9 Phylogenetic relationships, gene structure and expression pattern analysis of *PheMYB4* and *PheMYB4-1*. **(A)** Phylogenetic tree of *PheMYB4-1* and MYB proteins in selected plant species. The phylogenetic tree was constructed based on amino acid sequences using a neighbor-joining method **(B)** A multiple sequence alignment of *PheMYB4-1*, PheMYB4, *OsMYB4*, *AtMYB15*. The alignment was performed using ClustalX 2.1. A black background indicates conserved residues among all the proteins selected. The dashes indicate gaps introduced for better alignment. **(C)** The expression patterns of PheMYB4 and *PheMYB4-1* under ABA, MeJA and SA treatments in moso bamboo.

FIGURE S10 Expression and phenotypic analysis of *PheMYB4-1* transgenic *A. thaliana* plants. **(A)** Histogram showing the *PheMYB4-1* mRNA level normalized with respect to β-*Tubulin*. Different number of the asterisk represents the statistically different ^∗^*P* < 0.05; ^∗∗^*P* < 0.01. **(B)** Comparison of leaf size of WT and *PheMYB4-1* high expressing *Arabidopsis* line (HE-2). All the rosette leaves before bolting were carefully cut down from leaf base. **(C)** Photographs of the seeds shape and structure of WT, *PheMYB4-1* low expression lines (LE) and high expression line (HE-2). The deformed and shriveled seeds were mark out with a black circle and list separately at bottom, bar = 1 mm. **(D)** Morphology of severely deformed seeds in HE-2, bar = 1 mm.

FIGURE S11 Expression analysis of PheMYB4 and PheMYB4-1 under cold, drought and salt treatments in moso bamboo. Different number of the asterisk represents the statistically different ^∗^*P* < 0.05; ^∗∗^*P* < 0.01.

TABLE S1 Oligo nucleotide primer sequences used in qRT-PCR analysis.

TABLE S2 Nomenclature and Classification of MYB family genes in moso bamboo.

TABLE S3 The sequences of twenty conserved motifs in MYB proteins of moso bamboo.

TABLE S4 Expression profiles of *PheR2R3MYBs* during different stages of flower development.

TABLE S5 Statistical analysis of *PheMYB4-1*-overexpressing plants. Different lowercase letters represent the statistically different.
