# Supplementary figures and images for: The R2R3MYB Gene Family in Phyllostachys edulis: Genome-Wide Analysis and Identification of Stress or Development-Related R2R3MYBs
Source: Front Plant Sci. 2018 Jul 10;9:738. doi: 10.3389/fpls.2018.00738 (PMC6048295; doi:10.3389/fpls.2018.00738)

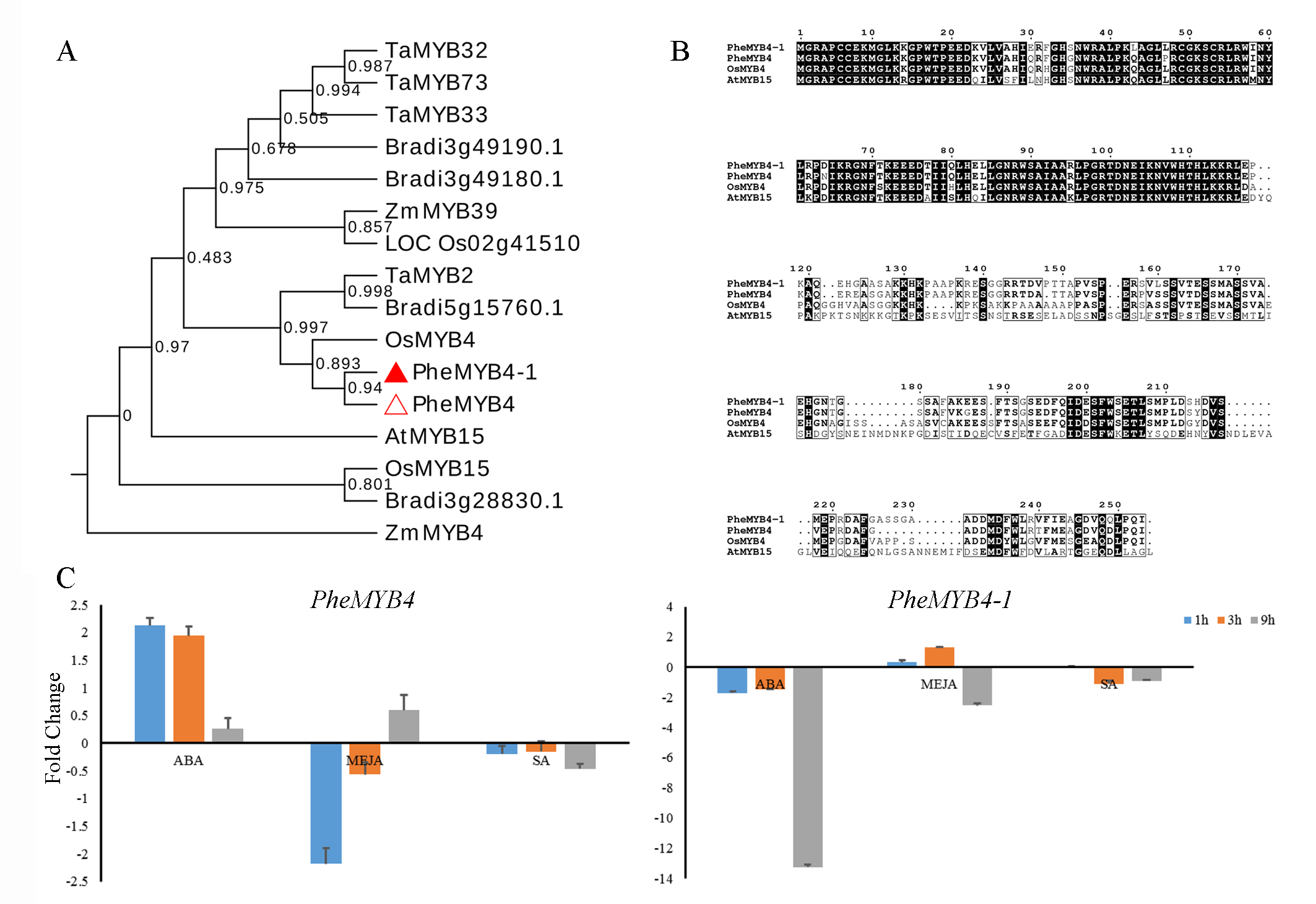

Supplement: FIGURE S1 — The maximum likelihood phylogeny tree of the MYB transcription factors of moso bamboo, Arabidopsis, rice, and Brachypodium. The colored shadow marks the subgroups of the MYBs. Numbers on branches are bootstrap proportions from 1000 replicates. [file Data_Sheet_1.ZIP › ▓╣│Σ▓─┴╧/FIG S9.tif]

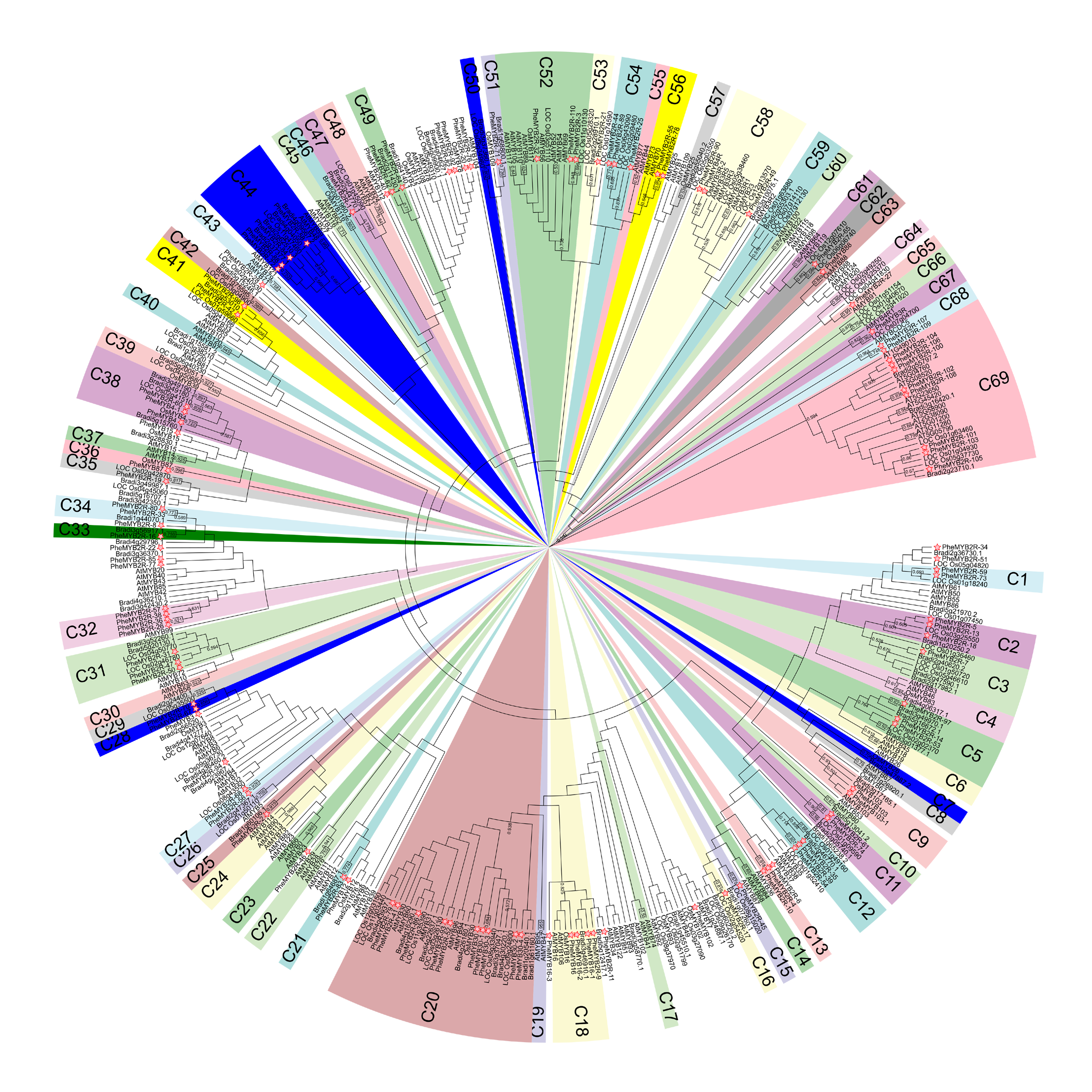

Supplement: FIGURE S1 — The maximum likelihood phylogeny tree of the MYB transcription factors of moso bamboo, Arabidopsis, rice, and Brachypodium. The colored shadow marks the subgroups of the MYBs. Numbers on branches are bootstrap proportions from 1000 replicates. [file Data_Sheet_1.ZIP › ▓╣│Σ▓─┴╧/Fig S1..tif]

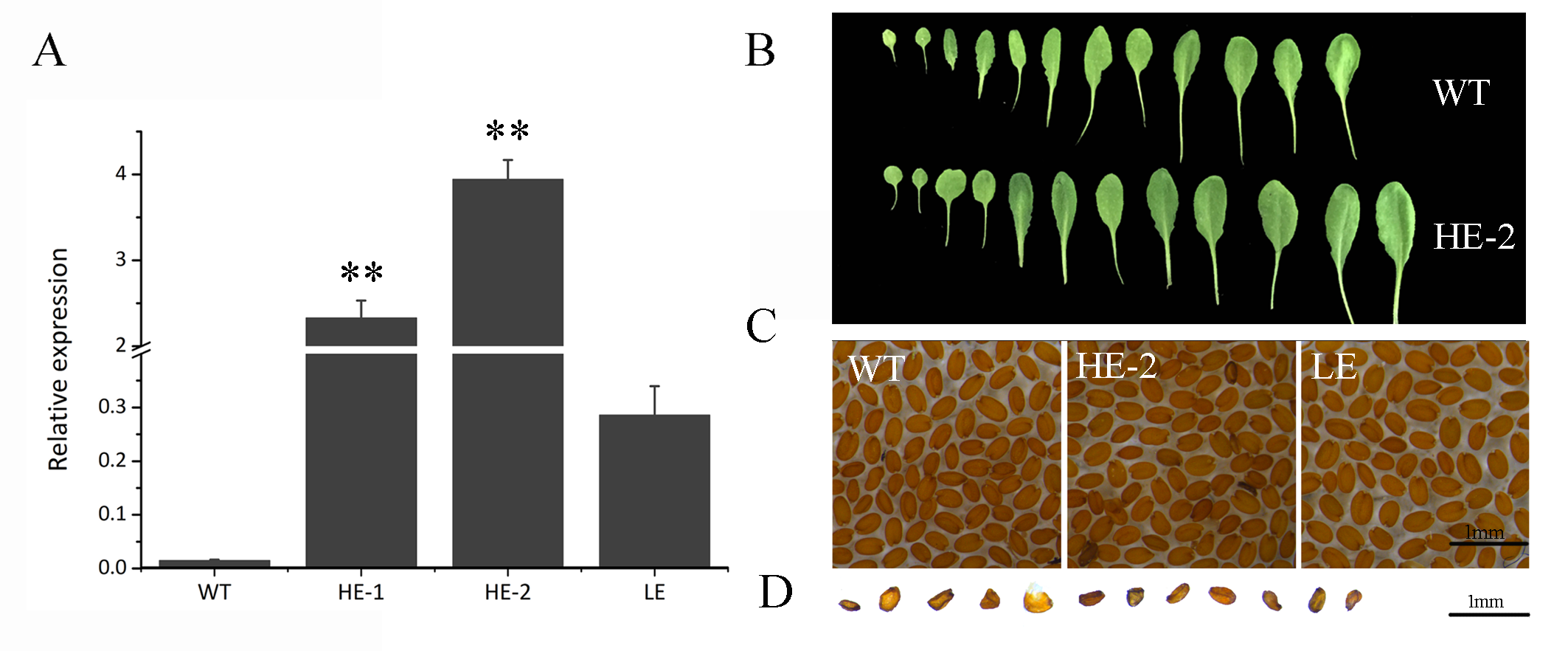

Supplement: FIGURE S1 — The maximum likelihood phylogeny tree of the MYB transcription factors of moso bamboo, Arabidopsis, rice, and Brachypodium. The colored shadow marks the subgroups of the MYBs. Numbers on branches are bootstrap proportions from 1000 replicates. [file Data_Sheet_1.ZIP › ▓╣│Σ▓─┴╧/Fig S10.tif]

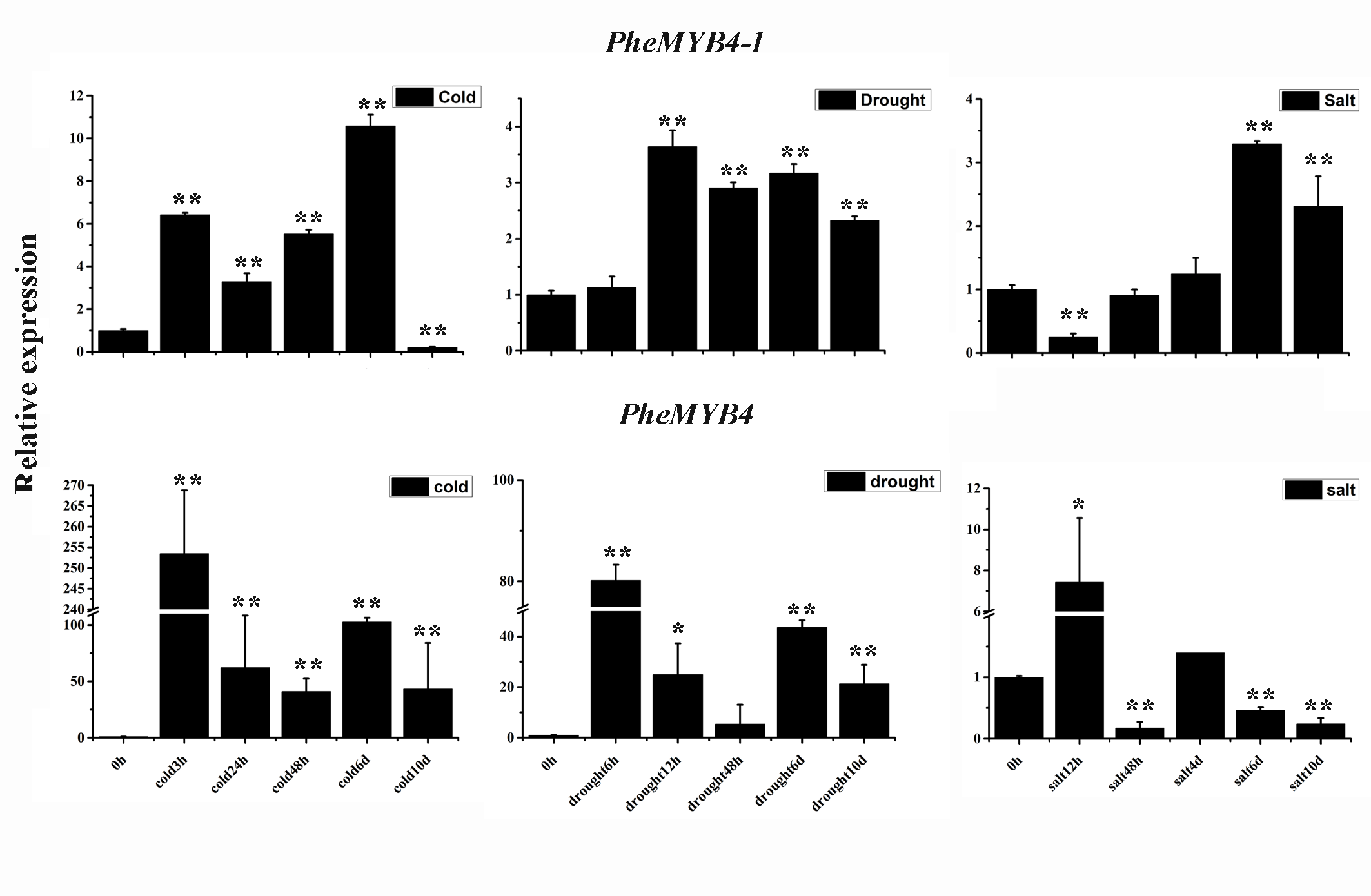

Supplement: FIGURE S1 — The maximum likelihood phylogeny tree of the MYB transcription factors of moso bamboo, Arabidopsis, rice, and Brachypodium. The colored shadow marks the subgroups of the MYBs. Numbers on branches are bootstrap proportions from 1000 replicates. [file Data_Sheet_1.ZIP › ▓╣│Σ▓─┴╧/Fig S11.tif]

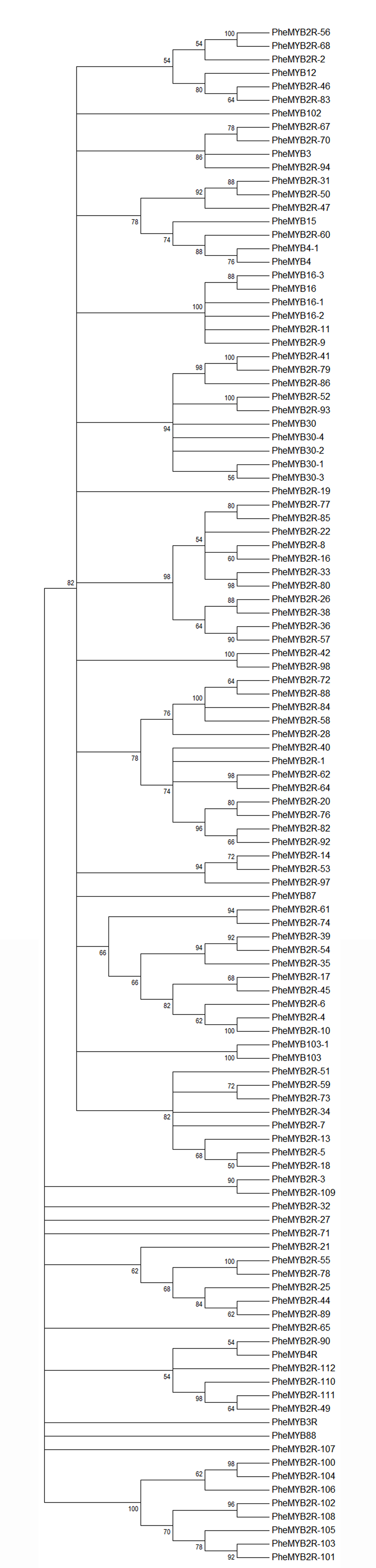

Supplement: FIGURE S1 — The maximum likelihood phylogeny tree of the MYB transcription factors of moso bamboo, Arabidopsis, rice, and Brachypodium. The colored shadow marks the subgroups of the MYBs. Numbers on branches are bootstrap proportions from 1000 replicates. [file Data_Sheet_1.ZIP › ▓╣│Σ▓─┴╧/Fig S2.tif]

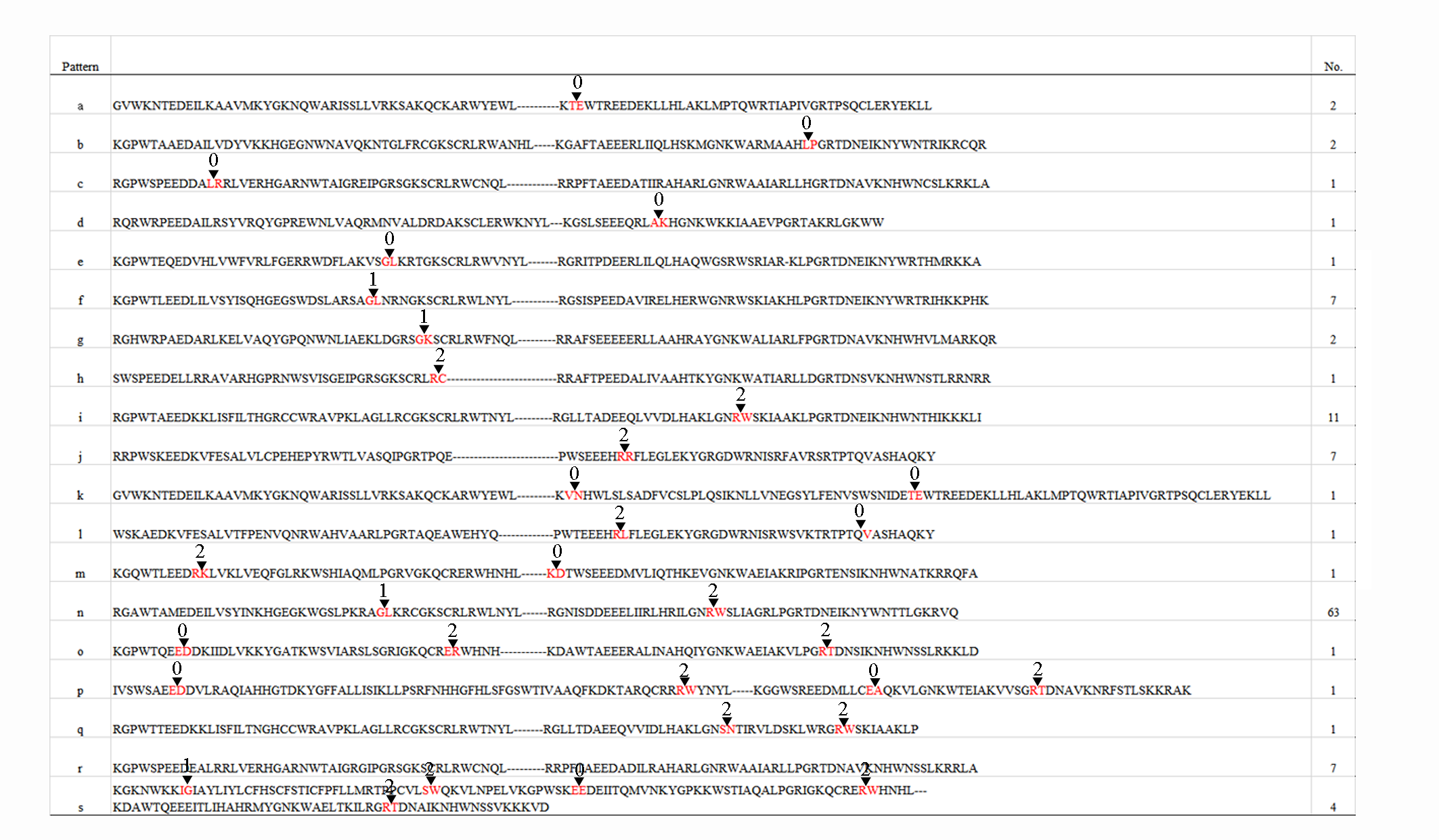

Supplement: FIGURE S1 — The maximum likelihood phylogeny tree of the MYB transcription factors of moso bamboo, Arabidopsis, rice, and Brachypodium. The colored shadow marks the subgroups of the MYBs. Numbers on branches are bootstrap proportions from 1000 replicates. [file Data_Sheet_1.ZIP › ▓╣│Σ▓─┴╧/Fig S3.tif]

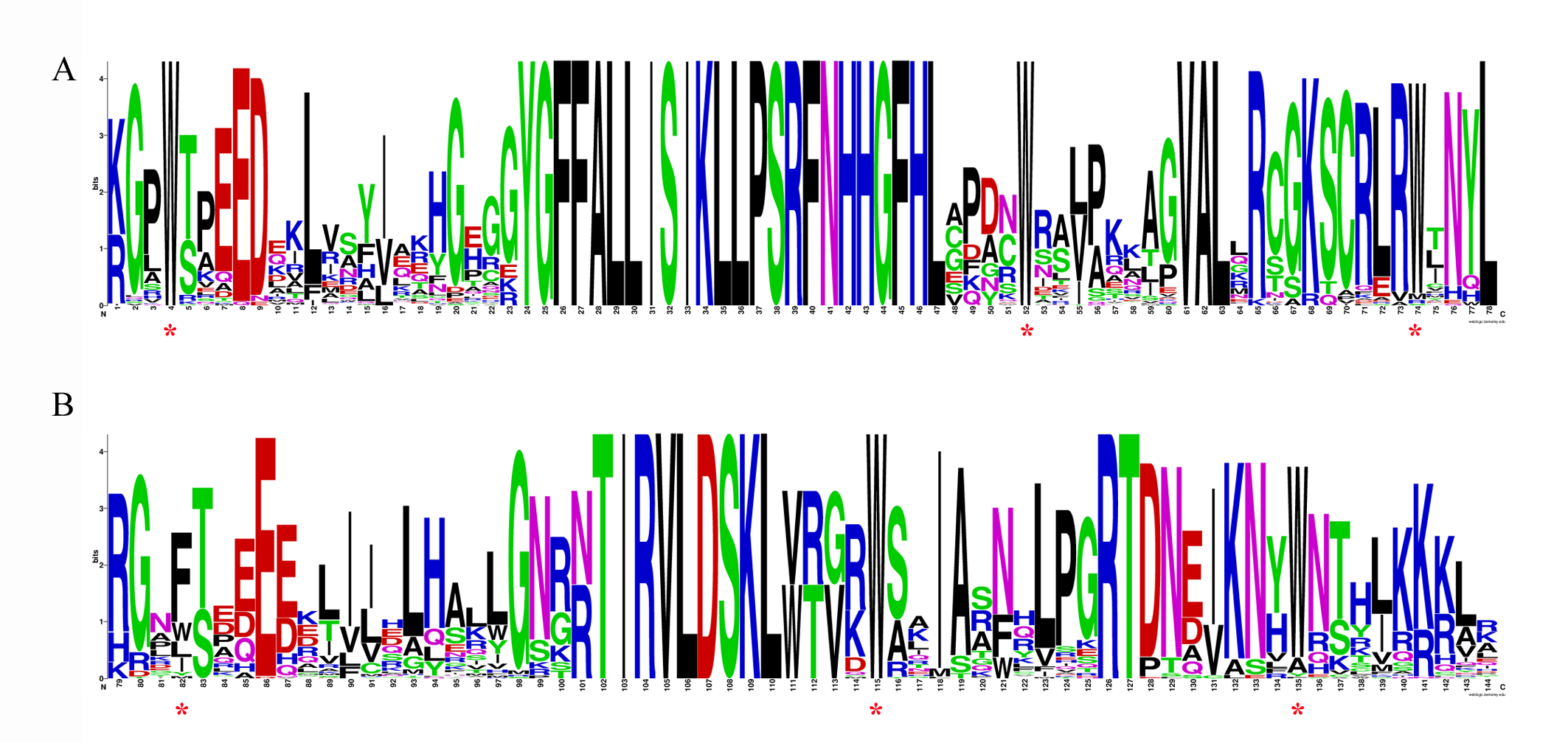

Supplement: FIGURE S1 — The maximum likelihood phylogeny tree of the MYB transcription factors of moso bamboo, Arabidopsis, rice, and Brachypodium. The colored shadow marks the subgroups of the MYBs. Numbers on branches are bootstrap proportions from 1000 replicates. [file Data_Sheet_1.ZIP › ▓╣│Σ▓─┴╧/Fig S4. The R2 and R3 MYB repeats across all the R2R3MYB proteins of moso bamboo .tif]

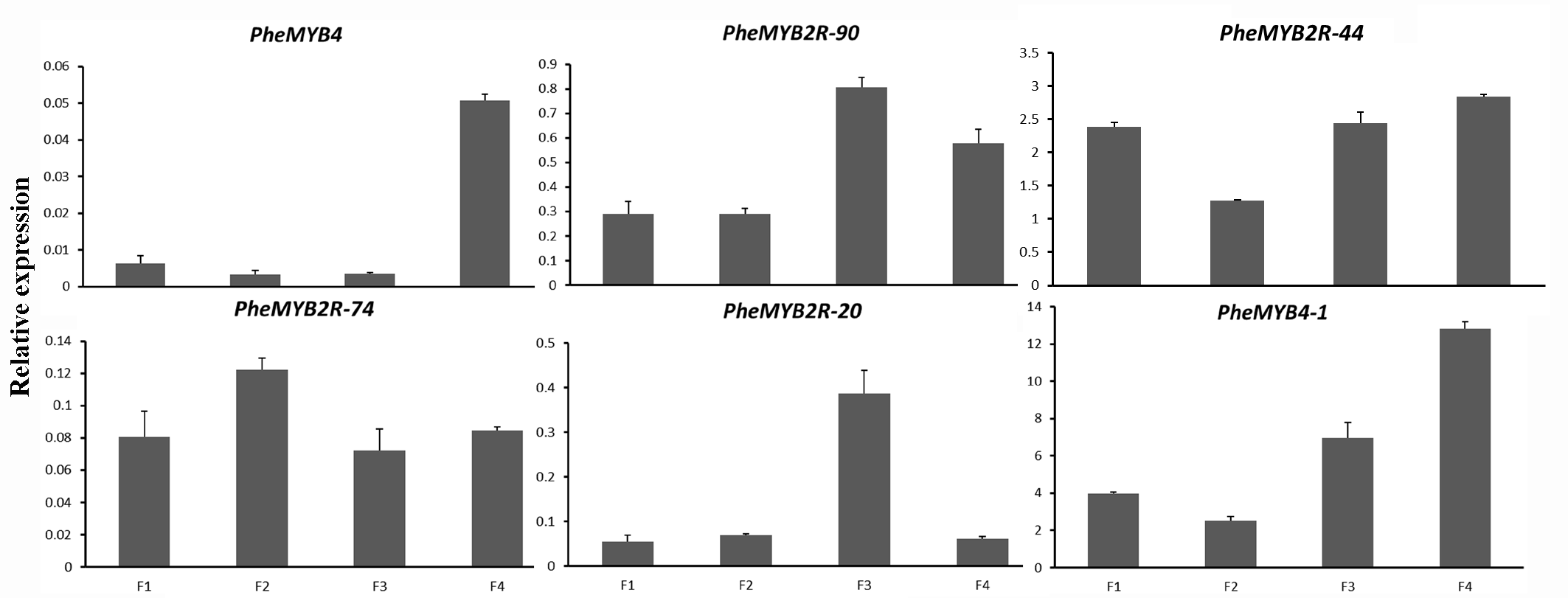

Supplement: FIGURE S1 — The maximum likelihood phylogeny tree of the MYB transcription factors of moso bamboo, Arabidopsis, rice, and Brachypodium. The colored shadow marks the subgroups of the MYBs. Numbers on branches are bootstrap proportions from 1000 replicates. [file Data_Sheet_1.ZIP › ▓╣│Σ▓─┴╧/Fig S5.tif]

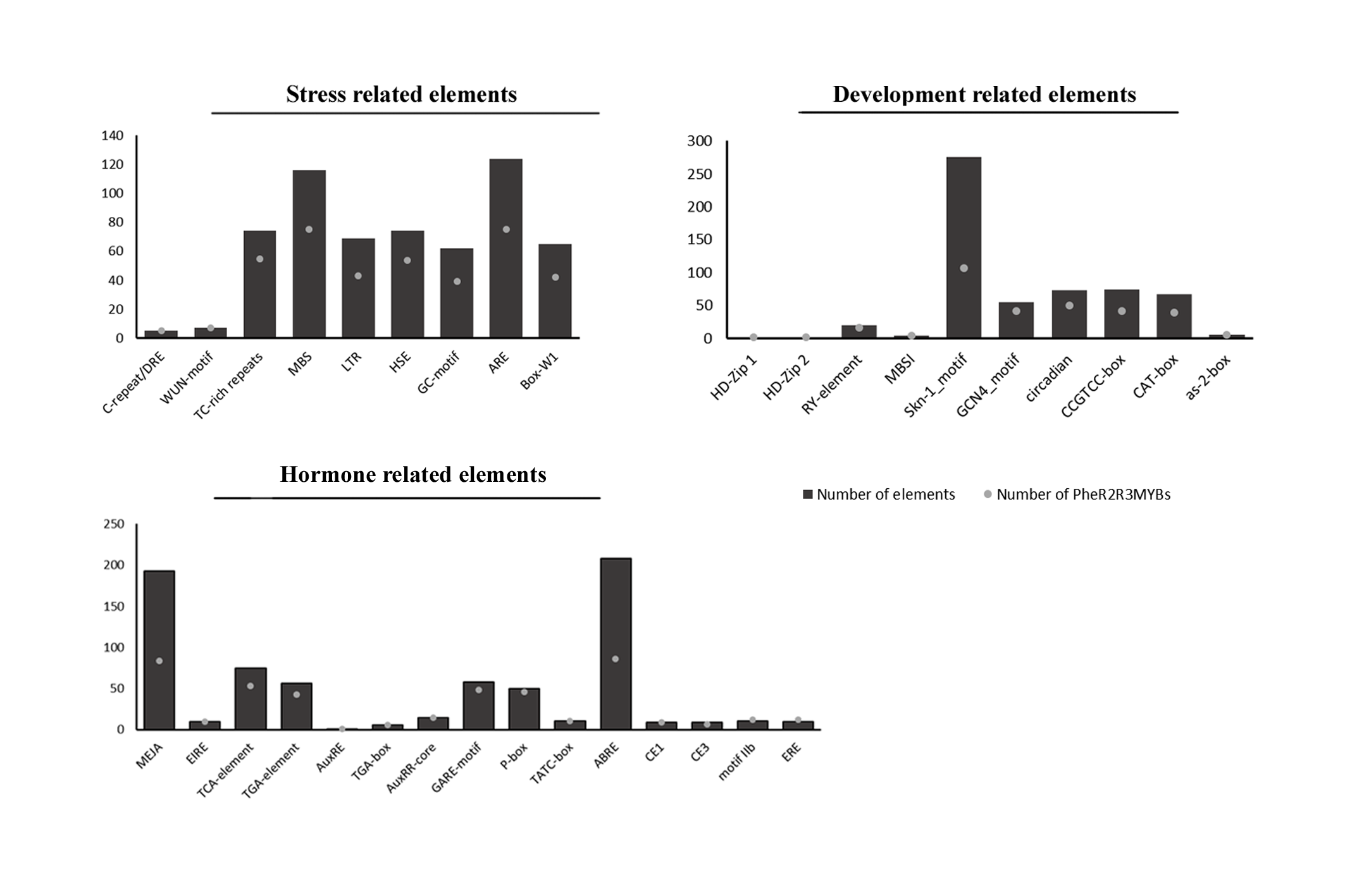

Supplement: FIGURE S1 — The maximum likelihood phylogeny tree of the MYB transcription factors of moso bamboo, Arabidopsis, rice, and Brachypodium. The colored shadow marks the subgroups of the MYBs. Numbers on branches are bootstrap proportions from 1000 replicates. [file Data_Sheet_1.ZIP › ▓╣│Σ▓─┴╧/Fig S6.tif]

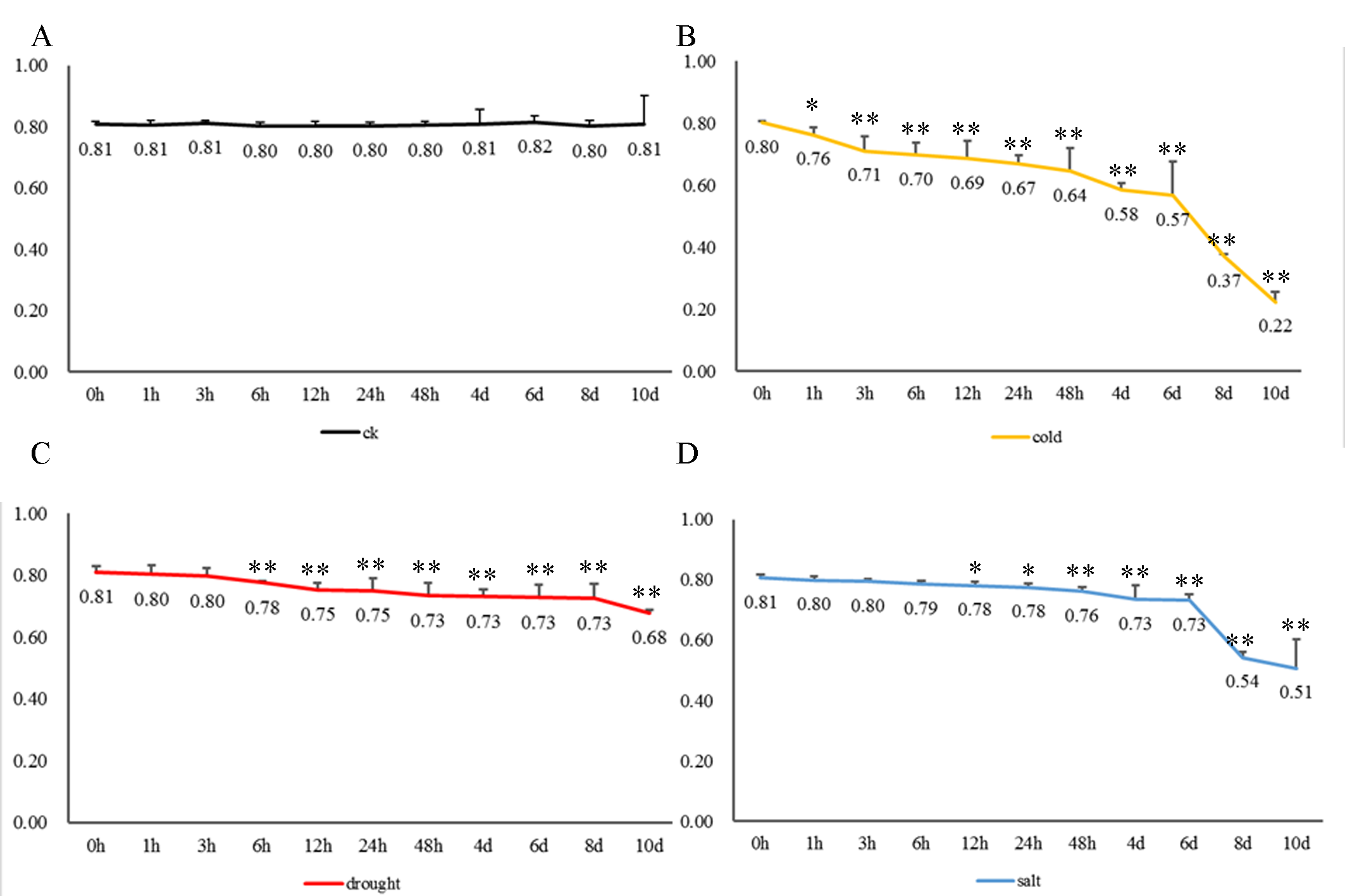

Supplement: FIGURE S1 — The maximum likelihood phylogeny tree of the MYB transcription factors of moso bamboo, Arabidopsis, rice, and Brachypodium. The colored shadow marks the subgroups of the MYBs. Numbers on branches are bootstrap proportions from 1000 replicates. [file Data_Sheet_1.ZIP › ▓╣│Σ▓─┴╧/Fig S7.tif]

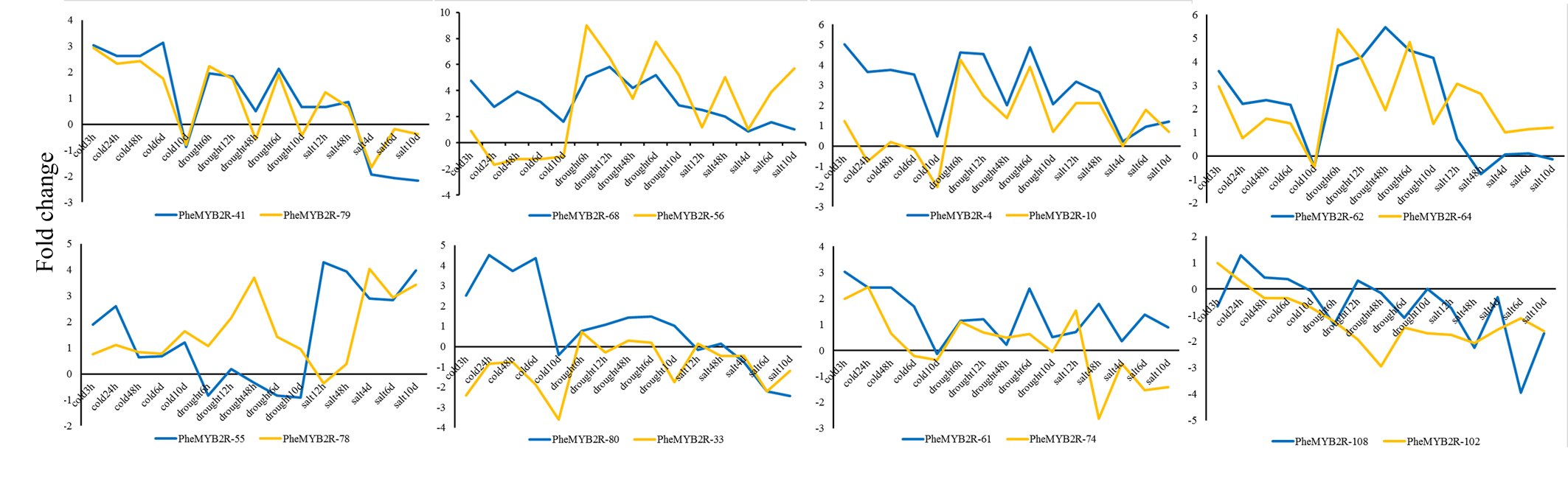

Supplement: FIGURE S1 — The maximum likelihood phylogeny tree of the MYB transcription factors of moso bamboo, Arabidopsis, rice, and Brachypodium. The colored shadow marks the subgroups of the MYBs. Numbers on branches are bootstrap proportions from 1000 replicates. [file Data_Sheet_1.ZIP › ▓╣│Σ▓─┴╧/Fig S8.tif]
